# Developmental and evolutionary comparative analysis of a regulatory landscape in mouse and chicken

**DOI:** 10.1242/dev.200594

**Published:** 2022-06-30

**Authors:** Aurélie Hintermann, Isabel Guerreiro, Lucille Lopez-Delisle, Christopher Chase Bolt, Sandra Gitto, Denis Duboule, Leonardo Beccari

**Affiliations:** 1 Department of Genetics and Evolution, University of Geneva, 30 quai Ernest-Ansermet, 1211 Geneva, Switzerland; 2 Swiss Institute for Experimental Cancer Research (EPFL ISREC), School of Life Sciences, Federal School of Technology (EPFL), 1015 Lausanne, Switzerland; 3 Collège de France, 11 Place Marcelin Berthelot, 75005 Paris, France

**Keywords:** Chromatin topology, TADs, Enhancers, Evolution, Gene regulation, Development, Teguments, Placodes

## Abstract

Modifications in gene regulation are driving forces in the evolution of organisms. Part of these changes involve *cis-*regulatory elements (CREs), which contact their target genes through higher-order chromatin structures. However, how such architectures and variations in CREs contribute to transcriptional evolvability remains elusive. We use Hoxd genes as a paradigm for the emergence of regulatory innovations, as many relevant enhancers are located in a regulatory landscape highly conserved in amniotes. Here, we analysed their regulation in murine vibrissae and chicken feather primordia, two skin appendages expressing different Hoxd gene subsets, and compared the regulation of these genes in these appendages with that in the elongation of the posterior trunk. In the two former structures, distinct subsets of Hoxd genes are contacted by different lineage-specific enhancers, probably as a result of using an ancestral chromatin topology as an evolutionary playground, whereas the gene regulation that occurs in the mouse and chicken embryonic trunk partially relies on conserved CREs. A high proportion of these non-coding sequences active in the trunk have functionally diverged between species, suggesting that transcriptional robustness is maintained, despite considerable divergence in enhancer sequences.

## Abstract (precis)

**Summary:** Analyses of the relationships between chromatin architecture and regulatory activities at the *HoxD* locus show that ancestral transcription patterns can be maintained while new regulations evolve.

## INTRODUCTION

Changes in the spatial and temporal regulation of genes crucial for developmental processes have greatly contributed to the evolution of animal morphologies (reviewed by Carroll, 2008; Rebeiz et al., 2015). The expression of such genes is generally modulated by combinations of *cis-*regulatory elements (CREs), short DNA sequences enriched for transcription factor binding sites, which tend to evolve more rapidly than the genes they control (reviewed by Edwards et al., 2013; Long et al., 2016; Spitz and Furlong, 2012). CREs can be spread over large distances around the gene(s) they regulate, which highlights the importance of the spatial organization of chromatin. Indeed, the existence of such ‘regulatory landscapes’ (Spitz et al., 2003) implies that enhancer-promoter interactions involve spatial proximity, which is generally thought to occur through large chromatin loops (Rao et al., 2014).

The extent of regulatory landscapes often correspond to topologically associating domains (TADs) (Andrey et al., 2013; Harmston et al., 2017), which were defined as chromatin domains in which the probabilities of interactions, as measured by chromosome conformation capture (Belton et al., 2012), are higher than those of the neighbouring regions (Dixon et al., 2012; Nora et al., 2012; Sexton et al., 2012). The distribution of TADs tends to be conserved in various vertebrate species, a phenomenon likely associated with regulatory constraints exerted by the complex and pleiotropic regulations found around many vertebrate developmental genes. Such domains, in which many interactions might occur, were proposed to form chromatin niches, rich in various DNA-binding proteins and in which the evolutionary emergence of novel regulatory sequences could be favoured due to the presence of both a pre-existing scaffold and appropriate co-factors (Darbellay and Duboule, 2016). Although genomic rearrangements that altered the structure of TADs were reported to impact proper gene regulation during development, sometimes leading to genetic syndromes (Bolt et al., 2021; Bompadre and Andrey, 2019; Lupiáñez et al., 2015), these rearrangements also affected enhancer-promoter spatial relationships in most cases, which makes a causal assessment difficult.

Genome-wide analyses carried out in different species, comparing DNA sequence conservation, TAD organization or some epigenetic modifications, have started to reveal some of the molecular mechanisms underlying both the evolution of gene regulation and, by extension, the modification of species-specific traits. However, integrated functional approaches are required to better understand how changes in chromatin architecture and modifications in CREs may influence the evolvability and the robustness of gene transcription. To address such questions, we used the *HoxD* locus as a paradigm of pleiotropic regulation of a multi-gene family, with a strong impact both on the control of important developmental steps and on the emergence of evolutionary novelties. The *HoxD* cluster contains a series of genes in *cis*, which encode transcription factors that are expressed in different combinations across several embryonic structures. The cluster is flanked by two large TADs, each one matching a gene desert highly enriched in CREs (Amândio et al., 2020; Darbellay and Duboule, 2016). These two TADs are separated by a boundary region which is localized within the cluster, close to its centromeric extremity (Andrey et al., 2013; Rodríguez-Carballo et al., 2017). The telomeric TAD comprises a large number of enhancers controlling transcription of various Hoxd genes in most of the tissues where HOX proteins operate (Andrey et al., 2013; Darbellay et al., 2019; Delpretti et al., 2013; Guerreiro et al., 2016; Schep et al., 2016). In contrast, the centromeric TAD contains enhancers specific for the transcription of genes involved in the development of terminal structures such as digits or external genitals (Amândio et al., 2020; Montavon et al., 2011).

Amongst the many tissues in which Hoxd genes exert their functions are the embryonic primordia of different skin derivatives, including those of mammary glands, vibrissae and pelage hairs of mammals, as well as those of chicken feathers (Kanzler et al., 1997; 1994; Reid and Gaunt, 2002; Reynolds et al., 1995; Schep et al., 2016). Despite the fact that these ectodermal structures share common developmental mechanisms and gene regulatory networks such as the Wnt, EDA and BMP signalling pathways (Biggs and Mikkola, 2014; Dhouailly et al., 2019; Di-Poï and Milinkovitch, 2016; Pantalacci et al., 2008; Wu et al., 2004), each type of primordium expresses different subsets of contiguous Hoxd genes. Because the specific topological organization of the *HoxD* locus can be observed in fishes (Acemel et al., 2016; Woltering et al., 2014), it likely predates the emergence of hairs and feathers during amniote evolution (Dhouailly, 2009; Wu et al., 2004). For this reason, these organs are valuable model systems to understand how CRE evolution within the pre-existing chromatin architecture could lead to the implementation of new gene-specific expression patterns. In contrast, the overall general organization of Hoxd gene expression along the main embryonic body axis is maintained across vertebrate species, despite low sequence conservation, raising the question as to how evolving Hox clusters could maintain globally similar transcriptional outputs.

Here, we addressed these issues by taking a comparative look at a well-defined regulatory landscape, positioned telomeric to the *HoxD* gene cluster, in mammals and in birds. These two syntenic regions contain CREs involved either in the same regulatory tasks or in controlling regulatory aspects specific to each taxon. In the latter context, we characterized Hoxd gene regulation in the mouse-specific vibrissae primordia (VPs) and in the chicken-specific feather primordia (FPs). We show that different subsets of contiguous Hoxd genes are expressed in the mouse VP mesoderm and in the chicken FP ectoderm, indicating an independent co-option of Hoxd paralogues in these structures. Although the transcription of these genes in VPs and FPs relies on lineage-specific CREs located within different segments of the regulatory landscape, these CREs exploit a largely conserved three-dimensional (3D) chromatin architecture.

Our comparative analysis of the gene regulation during trunk extension also revealed that *Hoxd1* displays an expression pattern markedly different from that of its neighbouring paralogues, both in mouse and chick, and that this *Hoxd1*-specific expression is driven by an evolutionarily conserved CRE located in a region predominantly interacting with *Hoxd1* in both species. However, the cis-regulatory code involved in *Hoxd1* gene regulation differs between the two species, suggesting that inter-species conservation of both gene and chromatin architectures is compatible with high plasticity of the *cis*-regulatory sequences involved. Extrapolating this observation across the genome suggests that the divergence of regulatory activities at conserved CRE sequences is more frequent than their maintainance.

## RESULTS

### Hoxd gene expression differs between mammalian and avian skin appendages

**Fig. 1.**
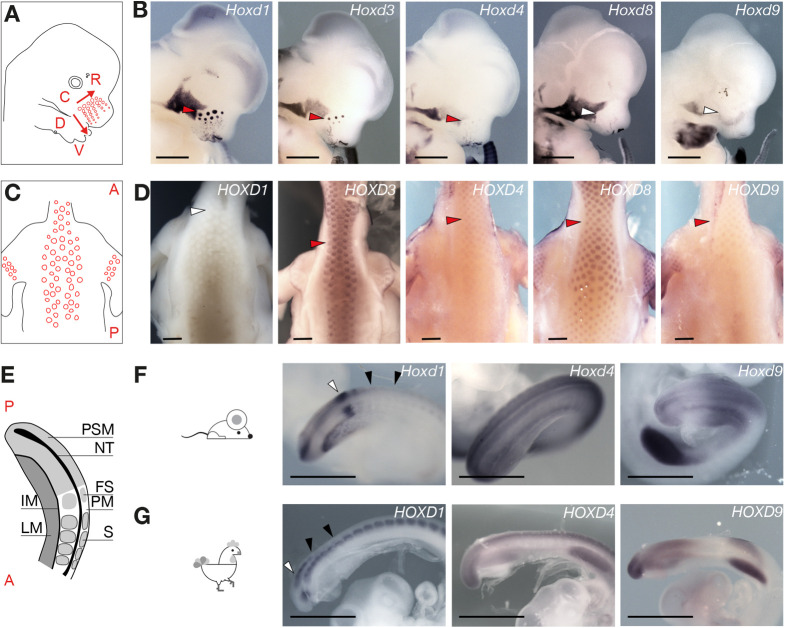
**Transcription of Hoxd genes in mammalian and avian skin primordia.** (A) Schematic of an E12.5 mouse embryo with vibrissae primordia (VPs) represented as red circles. The dorsoventral (D-V) and the caudorostral (C-R) axes are shown as red arrows, with the directions representing the timing of appearance of the VPs. (B) WISH on E12.5 faces. The arrowheads point to the most caudodorsal placode, which is the largest one and the first to appear. The red arrowheads indicate the detection of transcripts, white arrowheads indicate no detection of transcripts. Staining intensity progressively decreases from *Hoxd1* to *Hoxd8.* (C) Schematic of the back of a HH35 chicken embryo with feather placodes indicated as red circles. A, anterior; P, posterior. (D) WISH on HH35 chickens showing the skin of the upper back. The arrowheads point to the neck at the level of the shoulders. *HOXD1* is not expressed, whereas *HOXD3* and *HOXD8* signal intensities are stronger than for *HOXD4* and *HOXD9.* The red arrowhead indicates the detection of transcripts, white arrowheads indicate no detection of transcripts. (E) Schematic representation of a E9.5 or HH20 posterior trunk and tailbud. A, anterior; P, posterior; FS, forming somites; IM, intermediate mesoderm; LM, lateral mesoderm; NT, neural tube; PM, paraxial mesoderm; PSM, presomitic mesoderm; S, somites. (F) WISH on E9.5 mouse posterior trunk and tail bud. *Hoxd1* transcription pattern is different from that of *Hoxd4* and *Hoxd9.* (G) WISH on HH20 chicken posterior trunk and tail bud. *HOXD1* transcription pattern is different from *HOXD4* and *HOXD9.* The white arrowheads in panels F and G point to the *Hoxd1* stripe in the PSM. The black arrowheads show the difference between mouse (F) and chicken (G) in the persistence of *Hoxd1* mRNAs in formed somites. Images are representative of at least three different embryos processed in at least two different WISH experiments. Scale bars: 1 mm.

We initially analysed the expression profiles of Hoxd genes in mouse VPs and chicken FPs (Fig. 1). Skin appendage development starts with the interaction of the skin ectoderm with its underlying mesoderm, resulting in the thickening of the epithelium into a placode and in the condensation of dermal cells into a papilla (Mikkola, 2007; Sawyer and Knapp, 2003; Widelitz and Chuong, 1999). VP development starts at early embryonic day (E) 12, forming a stereotyped array of eight columns and five rows of VPs (Wrenn and Wessells, 1984) (Fig. 1A). The epithelial placode subsequently invaginates and engulfs the dermal papilla, forming the follicle primordium (Fig. S1). Likewise, in birds, ectoderm-mesoderm interactions result in the evagination of feather buds around E7 [stage Hamburger–Hamilton (HH) 30-32 (Hamburger and Hamilton, 1951; Michon et al., 2007)] (Fig. S2).

Whole-mount *in situ* hybridization (WISH) revealed strong *Hoxd1* expression in the dermal papillae of the VPs (Fig. 1B; Fig. S1). Furthermore, a comparative analysis of VPs from single E12.5 embryos revealed that *Hoxd1* transcripts accumulate slightly after ectodermal placode formation, marked by the expression of *Shh* (Chiang et al., 1999) (Fig. S1). *Hoxd3* and *Hoxd4* are also expressed in VPs, although at much lower levels than *Hoxd1* (Fig. 1B; Fig. S1). Faint levels of *Hoxd8* mRNA were sporadically detected in the VPs, whereas *Hoxd9* to *Hoxd13* mRNAs were never observed in these structures (Fig. 1B; Fig. S1). *In situ* hybridization on cryostat sections confirmed that *Hoxd1* transcripts accumulated in the dermal mesenchyme of VPs, but not in the ectoderm-derived region of the follicle (Fig. S1). Therefore, *Hoxd1*, *Hoxd3* and *Hoxd4* are transcriptionally active in the dermal papillae of developing VPs, with *Hoxd1* being the main paralogue expressed in these structures.

WISH experiments in HH35 chicken embryos revealed expression of *HOXD3*, *HOXD4*, *HOXD8* and *HOXD9* in the FPs, whereas *HOXD1* and *HOXD11* mRNAs were not detected (Fig. 1C,D; Fig. S2). Cryostat sections indicated that HOXD gene expression in chicken FPs occurred in the follicle ectoderm (Fig. S2), in contrast with murine VPs. Different Hoxd genes thus operate in the dermal papillae of mouse VPs and in ectodermal placodes of chicken FPs, likely reflecting the implementation of different regulatory mechanisms in the two lineages.

### Hoxd gene expression in the mouse and chicken embryonic body axis

Hox genes are expressed in the paraxial and lateral mesoderm of the main embryonic axis, as well as in the neural tube, and display progressively more restricted expression domains depending on their position within the cluster (Gaunt et al., 1988). Comparative WISH analysis of mouse and chicken embryos revealed that *Hoxd1* expression patterns in the trunk were markedly similar between the two species, but were largely divergent from those of the 5′-located neighbouring Hoxd paralogues (Fig. 1E-G). Although *Hoxd4* was strongly and uniformly transcribed in the mouse paraxial and lateral mesoderm as well as in the neural tube, *Hoxd1* displayed a biphasic expression pattern. *Hoxd1* transcripts accumulated in the embryonic tailbud; however, they rapidly disappeared from the presomitic mesoderm and remained undetected in lateral mesoderm progenitors or in the neural tube (Fig. 1F). Instead, *Hoxd1* was specifically re-activated during somite condensation, possibly coupling the transcription of this gene to the segmentation clock (Dale and Pourquié, 2000; Zákány et al., 2001). Similar to the expression patterns in mice, chicken *HOXD1* expression differed from its neighbouring paralogues, even though its transcripts remained detectable in the formed somites all along the main body axis (Fig. 1G). These observations suggested that *Hoxd1* transcription in developing somites may depend on a specific set of CREs, which are different from the CREs controlling the neighbouring paralogues, and that this locus-specific transcription was evolutionarily conserved across amniotes.

### An evolutionarily conserved topology at the *HoxD* locus

**Fig. 2.**
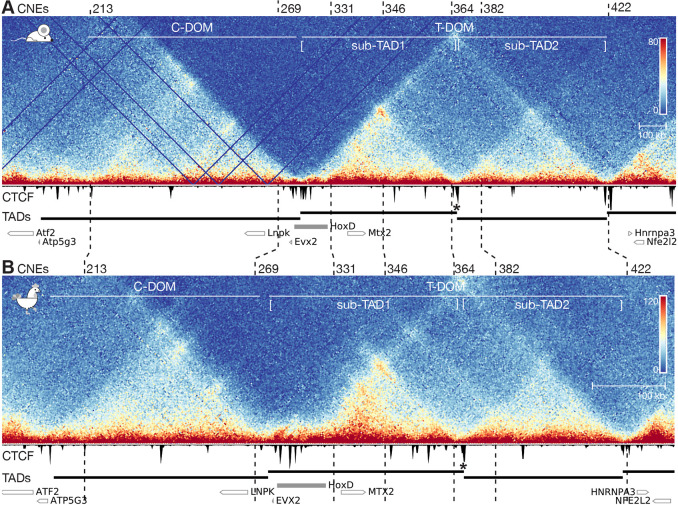
**Capture Hi-C-seq at the mouse and chicken *HoxD* loci.** (A,B) Capture Hi-C-seq heatmaps using dissected posterior trunk cells. Below each heatmap, a CTCF ChIP-seq track is shown, produced from the same material. The similarities in the structural organization of the mouse and the chicken loci are underlined either by the positions of syntenic CNEs at key positions (numbered vertical dashed lines), the domains produced by TAD calling (black bars below) and the presence of a sub-TAD boundary (asterisk) within T-DOM. Genes are represented by empty rectangles, the filled grey rectangle indicates the *HoxD* cluster. In panel A, E9.5 mouse posterior trunk cells were used and the CHi-C heatmap was mapped on mm10 with a bin size of 5 kb (chr2:73,800,000-75,800,000). In panel B, HH20 chicken posterior trunk cells were used and the CHi-C heatmap was mapped on galGal6 with a bin size of 2.5 kb (chr7:15,790,000-16,700,000) and on an inverted *x*-axis. The positions of the two TADs (T-DOM and C-DOM) and of both sub-TADs are shown on top. The scales on the *x*-axes were adjusted to comparable sizes for ease of comparison, yet the chicken locus is more compacted (scale bars are shown on the right).

In order to see whether these differences and similarities in regulatory specificities could be associated to variations in the global chromatin architecture at the *HoxD* locus, we performed Capture Hi-C (CHi-C; high throughput chromosome conformation capture in which the library is enriched for regions of interest by RNA probe hybridization) experiments using dissected mouse and chicken posterior trunks (Fig. 2A,B). Even though the global size of TADs at this locus is approximately two times smaller in the chicken genome (i.e. 1.7 Mb in mouse versus 0.8 Mb in chicken), the chicken *HOXD* region closely resembled its mouse counterpart, with the telomeric domain (T-DOM) being further organized into two sub-TADs in both species (Fig. 2, sub-TAD1 and sub-TAD2) (Rodríguez-Carballo et al., 2020; Yakushiji-Kaminatsui et al., 2018). Of note, the presence and relative distribution of conserved non-coding elements (CNEs) were maintained within this similar chromatin architecture (Fig. 2; Fig. S2D).

**Fig. 3.**
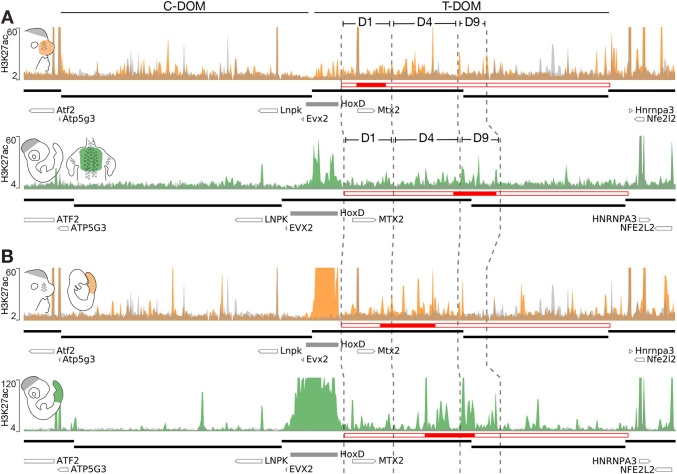
**Tissue- and gene-specific interactions of dense H3K27ac regions.** (A) The top panel shows a ChIP-seq profile of H3K27ac using mouse E12.5 VPs (orange) superimposed over the E12.5 forebrain cells (grey) (mm10, chr2:73,800,000-75,800,000). The lower panel shows a H3K27ac profile produced from dissected chicken HH35 dorsal skin (green), with HH18 brain cells (grey) (galGal6, chr7:15,790,000-16,700,000, inverted *x*-axis). The highest interacting regions are depicted as D1, D4 and D9 (see also Fig. S3B). The positions of conserved CNEs were used to delimit these regions in both species (vertical dashed lines) and the extents of TADs are shown below (thick black lines). The T-DOM (empty red rectangle) is split in overlapping genomic windows and the densest window (most acetylated region; MAR) is shown as a filled red rectangle. In the mouse VP, the MAR is within the D1 DNA segment, whereas the chicken MAR overlaps with the D4- and D9-regions*.* (B) The top panel shows a H3K27ac ChIP-seq using mouse E9.5 posterior trunk cells (orange) superimposed over forebrain cells (grey) (mm10, chr2:73,800,000-75,800,000). The lower panel shows the H3K27ac profile obtained from the same sample but dissected from a HH18 chicken embryo (green) with brain cells (grey) as a control (galGal6, chr7:15,790,000-16,700,000, inverted *x*-axis). Although the mouse posterior trunk MAR (red rectangle) coincides with the D4 segment, the chicken MAR counterpart also covers D4 and a small part of D9.

We corroborated these observations by assessing the interactions established by the *Hoxd1*, *Hoxd4* and *Hoxd9* promoters in mouse and chicken embryonic posterior trunk cells, using circularized chromosome conformation capture coupled with high-throughput sequencing (4C-seq) (Fig. S3). As an indicator for non-tissue-specific interactions, we used embryonic brain cells, in which Hoxd genes are not transcribed. In both species, the *Hoxd1*, *Hoxd4* and *Hoxd9* viewpoints established frequent contacts with the T-DOM, whereas interactions with the centromeric domain (C-DOM) were virtually absent. In addition, all viewpoints contacted T-DOM sub-TAD1 with a higher probability than T-DOM sub-TAD2, showing specific and non-overlapping regions where interactions were enriched. *Hoxd1* contacted the first half of sub-TAD1 (D1-region; Fig. 3A; Fig. S3) more intensively than *Hoxd4* or *Hoxd9* (Fig. S3)*.* In turn, *Hoxd4* contacts within the 3′ half of sub-TAD1 (D4-region; Fig. 3A; Fig. S3) were increased to the sub-TAD boundary (Fig. S3). Finally, *Hoxd9* interactions were mostly limited to the sub-TAD boundary region (D9-region; Fig. 3A; Fig. S3), where they were higher than for *Hoxd1* and *Hoxd4*. This distribution of interacting regions within the 3′ TAD were found in both brain and posterior trunk cells, albeit with an overall lower contact frequency in brain cells, reflecting the transcription-independent default conformation of the region, in agreement with previous reports (Andrey et al., 2013; Dekker and Heard, 2015; Noordermeer et al., 2011).

The regions of preferential interactions were annotated using a combination of CNE positions and 4C-seq coverage comparisons (Fig. S3C,D). The centromeric border of D1-region was set to CNE331, which is the CNE that is the most proximal from the *HoxD* cluster, but within the regulatory landscape. To define the boundary between the D1- and D4-regions and between the D4- and D9-regions, we used the coordinates for which the cumulative sum of the difference between the scores of two viewpoints was at its minimum, corresponding to the genomic location of the switch of preferential contacts from *Hoxd1* to *Hoxd4* and from *Hoxd4* to *Hoxd9*, respectively. Finally, the telomeric border of the D9-region was set to CNE382, which is located just after a clear, tissue-specific peak of *Hoxd9* contact frequencies.

Chromatin architectures partly rely upon the presence of the zinc-finger DNA-binding protein CTCF, which, together with the cohesin complex, can induce the formation and stabilization of large loops (Hansen et al., 2017; Nanni et al., 2020; Pugacheva et al., 2020). Chromatin immunoprecipitation sequencing (ChIP-seq) analysis of CTCF profiles revealed that both at the mouse and chicken *HoxD* loci, the telomeric end of the D1-region maps close to an occupied CTCF site (Fig. S3, blue arrowhead). Additionally, a cluster of CTCF-binding sites delimits the boundary between the mouse and the chicken sub-TAD1 and sub-TAD2 (Rodríguez-Carballo et al., 2017, 2020; Yakushiji-Kaminatsui et al., 2018), thus correlating with the limit between the D4- and D9-regions in both species (Fig. 2; Fig. S3, blue arrowheads in panels A,B).

Overall, these results show that *Hoxd1*, *Hoxd4* and *Hoxd9* preferentially interact with different regions of the T-DOM and that these segments are determined by the distribution of CTCF-binding sites in T-DOM subTAD-1. These distinct interaction patterns are constitutive and conserved between mouse and chicken. From these results, we concluded that the evolutionarily conserved contact topology of Hoxd genes with T-DOM may contribute to the implementation of their specific regulatory strategies in mouse and chicken embryonic structures.

### Tissue-specific regulatory sequences are located in regions of preferential interactions

To evaluate whether the promoter-specific partition of contacts with T-DOM is associated with the presence of differential regulatory activities, we proceeded to identify the CREs controlling Hoxd gene expression in the VPs, FPs and the embryonic trunk of both mouse and chicken embryos. We carried out ChIP-seq analyses using an antibody against H3K27ac, a histone modification indicative of an active chromatin state (Creyghton et al., 2010; Rada-Iglesias et al., 2011), and annotated tissue-specific regions with increased H3K27ac enrichment as most acetylated regions (MARs, Fig. 3). MARs were identified as genomic regions accounting for the highest H3K27ac enrichment after subtraction of the corresponding signal in brain cells (see Materials and Methods).

In the mouse VPs, the MAR was mapped within the D1-region (Fig. 3A, top, filled red rectangle), in agreement with the stronger expression of *Hoxd1* compared with its neighbouring paralogues (Fig. 1B; Fig. S1). However, in chicken dorsal skin samples in which *HOXD1* is not active (Fig. 1D), the MAR largely overlapped the D4- and D9-regions (Fig. 3A, bottom, filled red rectangle). Therefore, VPs and FPs display different distributions of H3K27ac enrichment across T-DOM, in agreement with the various subsets of Hoxd genes expressed in these tissues. In contrast, the MARs of both mouse and chicken posterior trunk samples largely overlapped the D4-region (Fig. 3B, filled red rectangles) as expected from their similar expression patterns in the embryonic trunk of both species, with the expression domain of *Hoxd4* being more extended than that of *Hoxd9* (Fig. 1F). In summary, the different enrichments of H3K27ac marks across T-DOM were located within the genomic segments that preferentially interacted with the genes expressed in each tissue.

### *Hoxd1* regulation in mutant regulatory landscapes

**Fig. 4.**
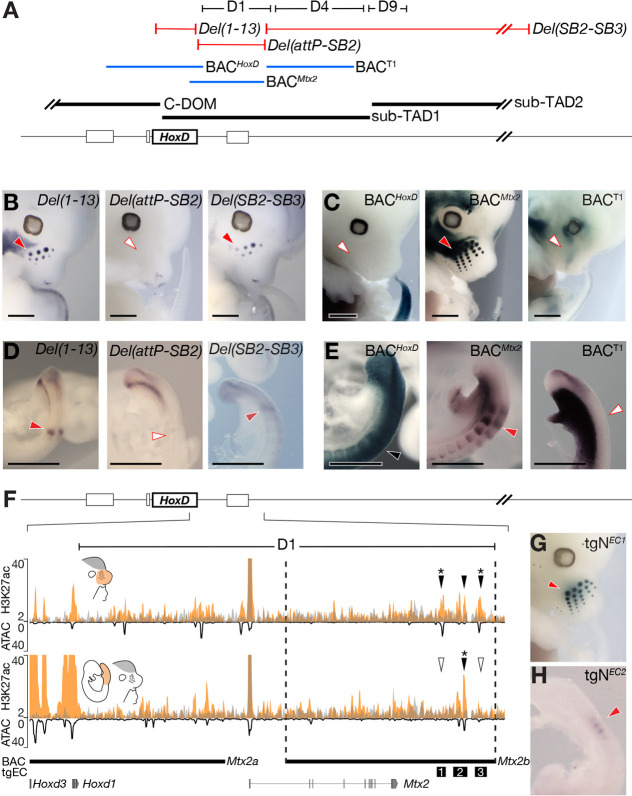
**Transcriptional regulation of *Hoxd1*.** (A) The three deletion lines used are shown in red and the transgenic BACs in blue. The extent of the D1- to D9-regions are shown on top (black) as well as the positions of both sub-TADs below (thick black lines). (B) WISH using a *Hoxd1* probe on E12.5 mouse embryos. *Hoxd1* mRNAs were detected in both the control and the *Del(SB2-SB3)* lines (red arrowheads), but the signal was absent from *Del(attP-SB2)* mutant embryos (white arrowhead). (C) X-gal staining of E12.5 mouse embryos carrying randomly integrated BACs. A strong staining was detected in VPs when BAC*^Mtx2^* was used (red arrowhead), whereas staining was not scored with the other two flanking BAC clones (white arrowheads). Therefore, a region directly downstream the *HoxD* cluster is necessary and sufficient to activate *Hoxd1* transcription in VPs, referred to as D1-region as it coincides with this previously defined region. (D) WISH of *Hoxd1* on E9.5 embryos focusing on tail buds. *Hoxd1* was detected in forming somites in both the control and the *Del(SB2-SB3)* lines (red arrowheads), but was absent from *Del(attP-SB2)* mutant embryos (white arrowhead). (E) X-gal staining of E9.5 mouse embryos carrying various BAC transgenes. Staining was scored in the entire neural tube and paraxial mesoderm with the BAC*^HoxD^* (black arrowhead). In contrast, embryos carrying BAC*^Mtx2^* displayed staining in forming somites (red arrowhead), a staining that was absent when BAC^T1^ was used (white arrowhead). (F) Enlargement of the D1-region along with a H3K27ac ChIP-seq using dissected E12.5 VPs (orange, top) and E9.5 posterior trunk cells (orange, bottom) superimposed over E12.5 forebrain (FB) cells (grey) (mm10; chr2:74,747,751-74,916,570). ATAC-seq using E12.5 VPs and E9.5 PTs are shown as a black line with an inverted *y*-axis to indicate levels of chromatin accessibility. Above the profiles are the three VP-acetylated elements (black arrowheads for H3K27ac-positive elements, asterisks for ATAC-positive elements) and below are MACS2 narrowPeaks for VPs and PTs (orange) and FB (grey). The position of the three EC sequences used as transgenes (tgEC) are shown as numbered black boxes. (G) X-gal staining of VPs from an E12.5 embryo transgenic (tgN) for *EC1*. (H) X-gal staining of forming somites in an E9.5 embryo transgenic for the *EC2* sequence. Red arrowheads in F,G indicate the detection of transcripts in VPs. White arrowheads indicate no transcript detection in VPs. Images are representative of at least two different embryos processed in at least two different WISH experiments. Scale bars: 1 mm.

To evaluate the functional importance of the D1-region, we assessed *Hoxd1* transcription in different mouse lines carrying mutations affecting this regulatory domain. We first analysed *Hoxd1* expression in the VPs of mouse embryos carrying various deletions of T-DOM (Fig. 4A, red lines). In the *HoxD^Del(attP-TpSB2)lac^* allele, the D1-region was removed [hereafter referred to as *Del(attP-SB2)*]. As *Del(attP-SB2)* homozygous embryos are not viable due to the deletion of the essential *Mtx2* gene (Andrey et al., 2013), we crossed this line with the mouse line *HoxD^Del(1-13)d9lac^* (Spitz et al., 2001) [hereafter referred to as *Del(1-13)*]. In the latter line, the entire *HoxD* cluster is deleted, in order to see the effect of the *HoxD^Del(attP-SB2)lac^* deletion on Hoxd gene regulation in *cis*. *Hoxd1* expression in the VPs of trans-heterozygous *HoxD^Del(attP-TpSB2)lac/Del(1-13)^* mutant embryos was completely abolished, in contrast with *Del(1-13)* heterozygous control littermates (Fig. 4B). Conversely, the *HoxD^Del(TpSB2-TpSB3)lac^* allele [hereafter *Del(SB2-SB3)*] (Andrey et al., 2013) carries a large deletion that encompasses the whole T-DOM except the D1-region. We detected robust *Hoxd1* expression in the VPs of E12.5 *HoxD^Del(TpSB2-TpSB3)lac/Del(1-13)^* trans-heterozygous mutant embryos (Fig. 4B).

We complemented these results by producing mouse transgenic embryos carrying bacterial artificial chromosomes (BACs) spanning the *HoxD* cluster and/or parts of sub-TAD1 (blue lines, Fig. 4A). In agreement with the analysis of the T-DOM deletions, the BACs covering either the *HoxD* cluster (BAC*^HoxD^*) (Schep et al., 2016) or mapping outside of the D1-region (BAC^T1^) (Delpretti et al., 2013) did not display any *lacZ* reporter activity in E12.5 to E13.5 VPs (Fig. 4C). Instead, mouse embryos that were transgenic for BAC*^Mtx2^* (Allais-Bonnet et al., 2021), which covers most of the D1-region, displayed strong reporter expression in the VPs (Fig. 4C). Notably, expression of *Hoxd1* was also abolished from facial mesenchymal progenitors in *Del(attP-SB2)* mouse embryos, whereas it was reported by X-gal staining of BAC*^Mtx2^* transgenic embryos, indicating that the D1-region regulates the transcription of *Hoxd1* in several tissues.

To assess whether the D1-region is also necessary and sufficient for the specific *Hoxd1* expression pattern in somites, we looked at *Hoxd1* transcription in posterior trunk cells using the same set of mutant alleles. *Hoxd1* expression was abolished in developing somites of *Del(attP-SB2)* E9.5 embryos compared with their control *Del(1-13)* littermates (Fig. 4A,D), whereas similar transcript levels were maintained in the tailbuds, suggesting that *Hoxd1* regulation in both structures involves different regulatory sequences. Accordingly, embryos transgenic for BAC*^Mtx2^* displayed strong *lacZ* expression in somites but not in the tailbud, with stronger activity in the last formed somites (Fig. 4A,E), thus closely mimicking native *Hoxd1* transcription in these structures (Zákány et al., 2001). In contrast, *lacZ* expression was uniform along the paraxial mesoderm of BAC*^HoxD^* transgenic embryos, without showing any *Hoxd1*-like specific reporter activity in the forming somites. Altogether, these results confirmed the regulatory potential of the mouse D1-region for *Hoxd1* expression patterns in facial tissues and in forming somites.

### Emergence of tissue-specific enhancers within conserved chromatin domains

*Hoxd1* transcription in VPs could have appeared through the evolution of new CREs, in association with the emergence of these structures in mammals. An alternative possibility would be the re-deployment of a pre-existing element already at work in somites. The latter hypothesis comes from the fact that in both VPs and somites, transcription of *Hoxd1* follows a cyclic dynamic, with a time course in phase with the production either of somites or of whisker pads. To gain insights into the evolution of *Hoxd1* regulation, we isolated individual VP enhancers to see if they would be active in somites. We generated two smaller BAC transgenes through homologous recombination of a *lacZ* reporter construct, starting with BAC*^Mtx2^*. Although BAC*^Mtx2a^* displayed no signal in VPs, BAC*^Mtx2b^* reported stable β-galactosidase activity (Fig. S4A). Within the BAC*^Mtx2b^* region, three candidate enhancers (ECs) were selected based on H3K27ac enrichment, two of them also being positive by assay for transposase-accessible chromatin with high-throughput sequencing (ATAC-seq) (Fig. 4F, black stars on arrowheads), thus corresponding to open chromatin regions in VPs. Of note, EC2 was ATAC negative in VPs, whereas H3K27ac was ATAC positive in posterior trunk cells (Fig. 4F, black and white arrowheads). The regulatory potential of the enhancer candidates was assessed using transient *lacZ* reporter assays in transgenic mouse embryos, and we identified a strong and reproducible enhancer activity of transgenic EC1 (*tgN^EC1^*) in VPs, comparable with that observed in embryos transgenic for BAC*^Mtx2^* (Fig. 4G). Instead, β-galactosidase activity was not detected either in *tgN^EC2^* or in *tgN^EC3^* transgenic embryos (Fig. S4B). We next tested these candidate enhancers at E9.5 to evaluate their regulatory potential in forming somites. *EC2*, which was the only candidate sequence with significant H3K27ac enrichment in posterior trunk cells, displayed reproducible activity in forming somites (Fig. 4H), unlike *tgN^EC1^* or *tgN^EC3^* (Fig. S4C). We concluded that the only VP enhancer sequence identified was not active during somite formation, unlike a somite enhancer located nearby (2.7 kb) within the D1-region.

The fact that two independent tissue-specific regulations rely on enhancers that are located in close proximity to a region of strong interactions suggests that a constrained topology may aid the emergence of novel regulatory sequences (Darbellay and Duboule, 2016). To investigate how enhancers might evolve within domains of preferential interactions and how their activities might relate to the observed transcriptional outputs, we compared our mouse and chicken H3K27ac and ATAC datasets with the levels of DNA sequence conservation. We analysed the number and distribution of putative enhancers (non-coding H3K27ac peaks overlapping with ATAC peaks) in the D1-, D4- and D9-regions of both mouse and chicken. CNEs were annotated by using BLASTZ pairwise alignments downloaded from the University of California, Santa Cruz (UCSC) Genome Browser and from which all elements overlapping with exons or promoters were discarded (Fig. S4D,E).

Overall, the number and distribution of putative enhancers in the different regions correlated with the transcriptional activity of Hoxd genes. A majority of putative enhancer sequences did not coincide with CNEs, suggesting a lineage-specific evolution of regulatory elements. Amongst those putative enhancers coinciding with CNEs, several displayed regulatory activities in one species only (Fig. S4, black arrows), indicating a divergence in the combinations of enhancers at work, even in posterior trunk cells, in which the transcriptional activity was expected to be similar in both species. This observation indicates that the sequence conservation of individual regulatory elements is not an obligatory requirement to maintain similar transcription patterns.

### Divergent enhancer activities of conserved sequences

**Fig. 5.**
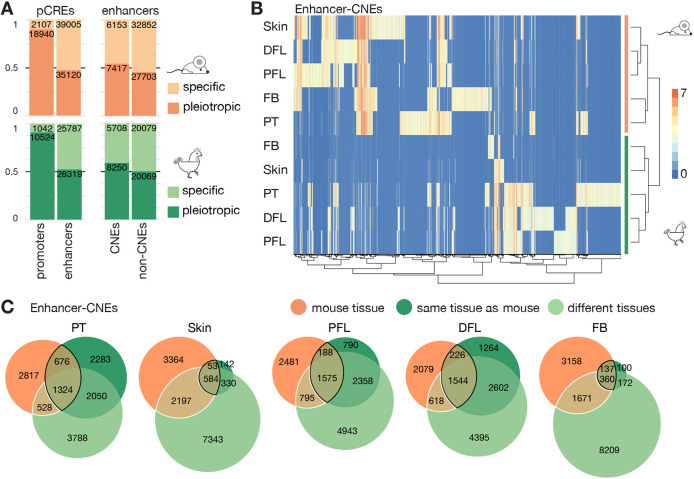
**Conserved sequences with divergent functions.** (A) Bar plots showing the proportion of putative *cis-*regulatory elements (pCREs) harbouring the H3K27ac mark in one (specific) versus more than one (pleiotropic) tissue, in either mouse (orange) or chicken (green). For simplification purposes, distal pCREs (>2 kb away from the transcription start site) are referred to as enhancers and proximal pCREs (<2 kb away from transcription start site) are referred to as promoters. The numbers of enhancers and promoters (left), and the numbers of conserved enhancers and non-conserved enhancers (right) are indicated. The proportion of specific versus pleiotropic elements is shown for CNE and non-CNE enhancers (right). In both mouse and chicken, enhancers are more specific than promoters and amongst enhancers, conserved elements are found to be acetylated in more tissues than non-conserved elements. (B) Hierarchical clustering obtained using the pheatmap R package. The *x-*axis indicates enhancer-CNEs, i.e. conserved sequences that are non-coding in mouse and in chicken and overlap with a H3K27ac mark. The *y-*axis shows the tissues used to obtain MACS2-processed peaks. The values on the heatmap correspond to the enrichment scores of the peaks. Enhancer-CNEs cluster by species and not by tissues. (C) Euler diagrams representing the numbers of enhancer-CNEs in the indicated tissues that are acetylated in mouse (orange), in the corresponding chicken tissue (dark green) or in unrelated chicken tissue (light green), showing that conserved sequences diverge in their regulatory activities. The intersection between the same tissue in mouse and in chicken is outlined in black, and the intersection between a chicken tissue different from the mouse tissue is outlined in white. DFL, distal forelimb; FB, forebrain; PFL, proximal forelimb; PT, posterior trunk.

To take this analysis genome-wide, we compared the H3K27ac profiles across different mouse and chicken embryonic tissues for the presence of CREs, by adding available proximal and distal forelimb datasets (Beccari et al., 2016; Yakushiji-Kaminatsui et al., 2018) to our posterior trunk and skin H3K27ac ChIP-seq datasets. Putative CREs (pCREs) were separated into putative enhancers and promoters, hereafter referred to as ‘enhancers’ and ‘promoters’ for simplicity, with ‘enhancers’ mapping more than 2 kb away from a gene transcription start site and ‘promoters’ mapping less than 2 kb away. pCREs which were H3K27ac positive in more than one of the analysed tissues were categorized as ‘pleiotropic’, whereas those being active in only one tissue were classified as ‘specific’. In both mouse and chicken, most promoters were pleiotropic, whereas the majority of the enhancers were specific (Fig. 5A), a result in agreement with the usual pleiotropic expression of genes carrying important developmental functions. When enhancers were divided into ‘CNEs’ or ‘non-CNEs’, i.e. those that displayed sequence similarities between mouse and chicken and those that did not, respectively, the proportion of pleiotropic enhancers was higher amongst the former (Fig. 5A; *P*<10^−15^ in mouse and in chicken, Fisher test), suggesting an increased level of sequence conservation that may reflect the higher evolutionary pressure on enhancers endorsing multiple regulatory functions.

Next, we used CNEs to compare the regulatory activities of murine and chicken orthologous sequences. In agreement with what was observed at the *HoxD* locus, the enhancer activities of CNEs, as inferred by H3K27ac peaks, more often diverged than they matched between the related mouse and chicken tissues (Fig. 5B,C). Hierarchical cluster analysis revealed that the enhancer profiles of CNEs were more similar between different tissues of the same species, than between the same tissues in mouse and chicken (Fig. 5B); a surprising observation as these CNEs were initially selected owing to their sequence conservation between the two species. We hypothesised that some sequences could have been conserved over time despite divergence in their regulatory activities because they would endorse other functions in other tissues. To evaluate this possibility, we used Euler diagrams to represent the relationships between mouse and chicken CNEs displaying enhancer features (Fig. 5C). Each mouse tissue was used as a reference in independent diagrams, in which chicken enhancer CNEs were represented as different sets, depending on whether they were active in the same or in different tissues compared with the mouse reference.

The intersection between the same tissue in mouse and in chicken represents elements that have both a conserved sequence and regulatory function (Fig. 5C, overlaps outlined in black). This intersection is smaller than any difference between two sets, which indicates that DNA sequence similarity between two species can be maintained amongst regulatory sequences despite divergence in their regulatory functions. We observed that CNEs often displayed enhancer activities in a chicken tissue that was different from the mouse tissue, supporting this idea (Fig. 5C, overlaps circled in white). Consequently, the regulatory activity of a given sequence conserved between these two species cannot be used to predict its function(s) in the two species.

To control for the technical specificity of our H3K27ac-based approach, we showed that chicken and mouse H3K27ac peaks reproducibly overlap ATAC-seq peaks. We first quantified the proportion of H3K27ac peaks that were also ATAC positive and found that most of them were indeed in an open chromatin state (Fig. S5A). We then repeated the enhancer-CNE quantifications to obtain ‘open enhancer CNEs’ by intersecting CNEs with open pCREs (Fig. S5). Again, open enhancer CNEs clustered by species (Fig. S5B), and the intersections on Euler diagrams were smaller than any difference between two sets (Fig. S5C). Therefore, the open pCRE quantifications recapitulated the results obtained using pCREs, thus supporting the validity of our H3K27ac approach.

Altogether, we show that both the global genomic organisation of the *HoxD* locus and specific domains of preferential enhancer-promoter interactions are highly conserved between mouse and chicken. However, the regulatory activities of individual CREs are largely species-specific and such sequences are systematically found within those segments of regulatory landscapes that preferentially interact with the promoters of the most highly transcribed genes. We also show that the lineage-specific evolution of regulatory activities could either generate new regulatory relationships or, conversely, maintain ancestral transcription patterns.

## DISCUSSION

### Versatile functions of Hoxd genes in skin appendages

Hox genes play a dual role in skin appendage development. Although their colinear expression is initially necessary to pattern the skin dermis (Kanzler et al., 1997), they are subsequently transcribed in the dermal papillae and/or ectoderm of embryonic and adult hair pelage follicles and vibrissae precursors (Godwin and Capecchi, 1998; Packer et al., 2000; Reynolds et al., 1995; Yu et al., 2018), as well as in the avian buds (Chuong et al., 1990; Kanzler et al., 1997). Here, we show that the dermal papillae of embryonic mouse vibrissae express *Hoxd1*, *Hoxd3* and *Hoxd4*, with the mRNA of *Hoxd1* being the first to be detected, followed by considerably lower levels of *Hoxd3* and *Hoxd4* mRNAs. The transcription in VPs is driven by CREs located in a region encompassing approximately 120 kb of DNA adjacent to the telomeric end of the mouse *HoxD* cluster, a region that is preferentially contacted by the *Hoxd1* gene. Therefore, a proximity effect is observed in the building of the chromatin architecture, based on constitutive contacts between *Hoxd1* and the neighbouring sub-TAD, whereby most of the *Hoxd1* regulatory sequences are located in the adjacent region of the regulatory landscape.

Although a detailed functional characterization of Hoxd genes during the development of whiskers was not the aim of this work, we did not observe any major morphological alteration in the vibrissae of mice carrying genetic deletions of this precise region, despite the complete abrogation in the expression of *Hoxd1*, *Hoxd3* and *Hoxd4* in these structures. This lack of a visible abnormal phenotype may be due to functional redundancy with other Hox paralogy groups expressed there, such as *Hoxc8* (Yu et al., 2018), a lack of analytical power or a lack of the appropriate behavioural paradigms that may have revealed functionally abnormal whiskers. The diverse expression of Hox paralogues in mammalian and avian skin appendages nonetheless illustrates the plasticity in the usage of Hox gene functions in these structures. This plasticity likely relies upon the independent acquisition of specific CREs for each gene cluster, as these skin derivatives emerged after the genome duplication events thought to be at the origin of the vertebrate lineage (Holland et al., 2008; Holland and Garcia-Fernàndez, 1996).

Although *Hoxd9*, *Hoxd11* and *Hoxd13* are expressed in the ectoderm-derived hair fibres (Godwin and Capecchi, 1998; Packer et al., 2000; Reynolds et al., 1995; Yu et al., 2018), we did not observe any *Hoxd1* in the vibrissae shaft, nor any *lacZ* expression when the BAC*^Mtx2^* (carrying the D1-region) transgenic animals were used, indicating that these expression patterns may depend on different sets of CREs. Our data also show that the expression of *HOXD3*, *HOXD4*, *HOXD8* and *HOXD9* in the ectoderm of the chicken FPs depends on CREs unrelated to those operating in the mouse VPs. These observations may indicate that different sets of Hoxd genes were independently co-opted during the evolution of skin derivatives, likely through *de novo* enhancer acquisition in the mammalian and avian lineages, in a way related to the regulation of *Hoxd9* and *Hoxc13* in the embryonic mammary buds and in the nails and hairs of mammals, respectively (Fernandez-Guerrero et al., 2020; Schep et al., 2016). Alternatively, the rapid pace of tegument evolution in tetrapods may have been accompanied by a divergence in enhancer sequences, which makes comparisons difficult. In this context, we cannot rule out the possibility that Hoxd gene regulation in skin appendages of both mammals and birds derives from an initial pan-Hoxd expression pattern both in the ectoderm and mesoderm of skin appendages, which then evolved differently in each lineage through modification of the CRE complement located within the adjacent TAD. The fact that the two CNEs acetylated in mouse VPs and chick FPs are also acetylated in mouse and chick posterior trunk cells may indicate that the former structures co-opted parts of the regulatory mechanisms at work in the latter. This indicates that DNA sequence conservation may be due to constraints imposed by the function in the trunk rather than by the function in the skin.

### Enhancer acquisition in evolving tetrapod skin appendages

TADs containing pleiotropic genes of key importance for vertebrate development are frequently conserved across tetrapods, and topological changes in their organization often correlate with important modifications in gene expression (Eres et al., 2019; Laverré et al., 2022; Torosin et al., 2020; Yakushiji-Kaminatsui et al., 2018). Our results show that despite a considerable divergence in the non-coding elements localized in the mouse and chicken T-DOM, the global pattern of interactions is conserved, leading to similar chromatin topologies. Indeed, in both cases, different Hoxd genes interact preferentially with distinct regions of sub-TAD1. These contacts are for the most part constitutive, even though we observed an activity-dependent increase in interactions in the mouse VPs and in mouse and chicken posterior trunk cells. Of interest, the regions within T-DOM (D1 to D9), which are preferentially contacted by the *Hoxd1*, *Hoxd4* or *Hoxd9* genes, are organized in a linear sequence opposite to the order of the genes themselves, as if *Hoxd1* was folding in towards its closest possible chromatin segment within the T-DOM, bringing along *Hoxd4* towards the next chromatin segment and positioning the interactions involving *Hoxd9* further away into the T-DOM. This sequential positioning of interactions in the reverse order of spatial colinearity may impose a generic contact pattern, whereby *Hoxd9*, for example, is unable to establish strong interactions with the D1-region and its enhancers, as this region is primarily contacting *Hoxd1*, thus imposing constraints upon the distributions of interactions (see below).

Furthermore, the distribution of H3K27ac-positive regions in the chicken FPs and mouse VPs across these interacting regions correlates well with the specific subsets of Hoxd genes expressed in each of these tissues. Finally, the regulation of *Hoxd1* in developing somites relies on evolutionarily conserved elements located in the D1-region. This general regulatory topology is close to that observed in teleost fishes (Acemel et al., 2016; Woltering et al., 2014), suggesting that it was already established at the root of the vertebrate lineage and that its hijacking by nascent enhancer sequences may have favoured the co-option of specific Hoxd gene subsets as targets of the novel regulations. The regulation of Hoxd genes in mouse VPs and chicken FPs, as well in mouse mammary buds (Schep et al., 2016), may illustrate this process.

### Plasticity of the *cis*-regulatory code versus conservation in TAD organization

Owing to their functional relevance in the regulation of gene expression, CREs tend to be evolutionarily conserved across species (Sandelin et al., 2004; Sanges et al., 2013; 2006). However, several studies have shown that enhancers controlling developmental gene expression can be functionally conserved even across distant phylogenetic lineages, despite a strong divergence in their DNA sequences (Ambrosino et al., 2019; Berthelot et al., 2018; Wong et al., 2020; Eichenlaub and Ettwiller, 2011; Schmidt et al., 2010; Villar et al., 2015). Our inter-species comparison of CNEs enriched in H3K27ac across different embryonic structures revealed that a considerable portion of evolutionarily conserved CREs diverged in their patterns of activity between the two species analysed, in agreement with previous observations (Dermitzakis and Clark, 2002; Schmidt et al., 2010; Vierstra et al., 2014; Villar et al., 2015). This dichotomy between the conservation of enhancer sequences and their function is illustrated by the CREs controlling *Hoxd1* expression in mouse and chicken developing somites. Although some of the sequences active in the mouse posterior trunk are evolutionarily and functionally conserved in chicken, such as EC2, which can drive *lacZ* expression in the last formed somites of the murine embryo, other elements conserved at the nucleotide level displayed H3K27ac enrichment only in the mouse.

In fact, a large fraction of H3K27ac-positive sequences detected either in mouse or in chicken posterior trunk cells were not conserved between the two species, suggesting that they evolved in a species-specific manner. In contrast, the high proportion of chicken promoters that display similar H3K27ac enrichments in mice, together with the similar expression patterns observed for mouse and chicken Hoxd genes in embryonic trunk cells, demonstrates that conserved gene expression can occur despite a high evolutionary plasticity in the *cis*-regulatory code usage, in agreement with previous reports (Berthelot et al., 2018; Snetkova et al., 2021). This may point to a regulatory strategy in which multiple enhancers coordinate to regulate the same target gene(s), thus creating a quantitative effect in a way similar to the reported action of super-enhancers (Sabari et al., 2018), yet in which various subsets of CREs may display distinct tissue-specificities. This process may be implemented preferentially within large regulatory landscapes, e.g. in the case of *Fgf8* (Marinic et al., 2013) or in the TAD controlling Hoxd gene transcription in external genitals (Amândio et al., 2020).

### A regulatory playground with constraints

In those cases that were investigated, the respective positions of regulatory sequences within large syntenic regulatory landscapes are generally conserved, and the global regulatory architecture found at one particular developmental locus in one species can be extrapolated to its cognate locus in another amniote species (Kragesteen et al., 2018), implying that enhancer-promoter interactions are also evolutionarily well conserved, even for CREs acting over long distances (Irimia et al., 2012; Laverré et al., 2022), although some mechanistic elements may vary (Ushiki et al., 2021). In agreement with this, and contrasting with the apparent divergence in the *cis*-regulatory code controlling Hoxd gene expression in mouse and chicken embryos, our phylogenetic footprinting and epigenetic analysis revealed that non-coding elements conserved in the DNA sequence between the mouse and chicken genomes were distributed across the two species in a very similar manner. Therefore, the potential gain and/or loss of particular CREs in the two lineages did not significantly impact the internal organization of those conserved regulatory elements, nor did they impact the global chromatin topology of the locus. This confirms the observation that even the significant divergence in the CREs located at the *HoxD* locus, which accompanied the evolution of the snake body plan, did not result in any substantial alteration of the overall corn-snake Hoxd gene interaction map (Guerreiro et al., 2016). Altogether, these observations suggest that the internal organization of the TADs at the *HoxD* locus and the specific interaction profiles for various subsets of Hoxd target genes are resilient to considerable variation in their CREs.

These results also highlight the somewhat dual properties that large regulatory landscapes may have imposed in the course of regulatory evolution. On the one hand, a pre-established chromatin topology, as materialized by TADs, may have provided unique ‘structural niches’ to evolve new enhancer sequences, owing to the proximity of factors already at work. This might especially apply to landscapes in which a strong quantitative parameter may be instrumental, and *a fortiori* when the target gene(s) are acting in combination, coding for proteins of rather low specificities (Bolt and Duboule, 2020). This may be illustrated nowadays by large landscapes, in which all kinds of enhancer sequences are mixed within the same TAD. On the other hand, although this regulatory playground may stimulate the emergence of regulations, it will constrain the realms of action of the enhancer sequences, due to both the very architecture that favours their evolution and the accessibility to a single subset of contiguous target genes, as a result of the global distribution of interactions at this precise place in the landscape.

## MATERIALS AND METHODS

### Mouse strains and chicken eggs

All mutant mouse strains are listed in Table S1 and were backcrossed continuously to Bl6/CBA mixed animals and maintained as heterozygous stocks. Chick embryos from a White Leghorn strain were incubated at 37.5°C and staged according to Hamburger and Hamilton (1951).

### Enhancer cloning and transgenic animals

The different CRE candidate sequences were amplified using PCR-specific primers (see Table S2) and the Expand High Fidelity PCR system (Roche). Amplified fragments were gel purified and cloned into the pSK-lacZ vector (GenBank X52326.1; Beccari et al., 2016). For the transgenesis assays, each *lacZ* reporter construct was digested with NotI and KpnI, and the restriction fragment encoding the potential enhancer sequence, the β-globin minimal promoter and the *lacZ* reporter gene were gel purified and injected into the male pronucleus of fertilized oocytes. F0 embryos were dissected either at E10 or at E12, fixed and stained for β-galactosidase activity according to standard protocols.

### *In situ* hybridization

The probes used in this study for *in situ* hybridization were either previously reported or produced by PCR amplification of a fragment subsequently ligated in pGEMT Easy vector (Promega). Probe sequences and references are listed in Table S3. Digoxigenin (Dig)-labelled probes were synthesized *in vitro* by linearizing the respective plasmids using specific restriction enzymes and transcribed *in vitro* with T3 (Promega, P2083), T7 (Promega, P2078) or Sp6 (Promega, P1085) RNA polymerase (Table S3) and the Dig-RNA labelling mix (Roche). The probes were purified using the RNeasy Mini Kit (QIAGEN, NC9677589). WISH experiments were performed as described in Woltering et al. (2009). *In situ* experiments in sections were performed according to the protocol of Sockanathan (2015; https://protocolexchange.researchsquare.com/article/nprot-3781/v1). Cryostat sections were prepared after fixing embryos in 4% paraformaldehyde solution at 4°C overnight. Subsequently, embryos were washed three times with PBS and passed to increasing sucrose solutions at 4°C in agitation (15% sucrose solution for 3-4 h, followed by incubation in 30% sucrose solution overnight) and finally included in OCT blocks (TissueTek O.C.T Compound 4583). Sections of 20 µm were obtained with a Leica CM 1850 cryostat and stored at −80°C.

### Capture Hi-C-seq experiments

The SureSelectXT RNA probe design and capture Hi-C experiments were performed as described by Bolt et al. (2021) for mouse and as by Yakushiji-Kaminatsui et al. (2018) for chicken. Dissected tissues were processed as in Yakushiji-Kaminatsui et al. (2018) with the following change: cells were crosslinked in 2% formaldehyde in PBS and washed three times instead of being quenched by glycine. Hi-C-libraries were prepared as in Yakushiji-Kaminatsui et al. (2018). The first part of the data analysis was performed on our local Galaxy server (Afgan et al., 2016). Raw reads were preprocessed with CutAdapt (v1.16) (-a AGATCGGAAGAGCACACGTCTGAACTCCAGTCAC -A AGATCGGAAGAGCGTCGTGTAGGGAAAGAGTGTAGATCTCGGTGGTCGCCGTATCATT --minimum-length=15 --pair-filter=any --quality-cutoff=30) (Martin, 2011). Then, Hicup (v0.6.1) (Wingett et al., 2015) and Samtools 1.2 (Danecek et al., 2021) were used with default parameters. The pairs were then loaded to 5 kb (mouse) and 2.5 kb (chicken) resolution matrices with Cooler v0.7.4 (Abdennur and Mirny, 2020). These matrices were balanced with HiCExplorer hicCorrectMatrix (v3.7.2) using ICE as the correction method, the mad threshold from HiCExplorer hicCorrectMatrix diagnostic plot as the minimum threshold value and five as the maximum threshold value. The TAD separation scores were computed with HiCExplorer hicFindTADs (Ramírez et al., 2018; Wolff et al., 2020) with a fixed window of 240 kb in mouse and 120 kb in chicken. See CHiC_processRaws.sh https://gitlab.unige.ch/Aurelie.Hintermann/hintermannetal2022 for details.

### ATAC-seq

Embryos were collected and placed into PBS with 10% fetal calf serum and 15 μl collagenase at 50 mg/ml (Sigma-Aldrich, C9697) at 37°C for approximately 10 to 15 min. Two replicates were done using one embryo each and 50,000 cells were isolated for processing with the Nextera Tn5 enzyme (Illumina, FC-131-1096) as previously described (Buenrostro et al., 2013). Tn5-treated DNA was amplified with Nextera Library primers using the NEBNext library amplification master mix (New England BioLabs, M0541) and sequenced on an Illumina NextSeq 500. Sequenced DNA fragments were processed as previously reported (Amândio et al., 2021) with the following minor modification on a local Galaxy server (Afgan et al., 2016): the BAM file was converted to BED prior to peak calling with bedtools version 2.30.0 (Quinlan and Hall, 2010). Peak calling was done using MACS2 (v2.1.1.20160309) callpeak (--no-model --shift −100 --extsize 200 --call-summits --keep-dup all). Peak regions for each condition were obtained from the union of the peak region of both replicates using bedtools merge (v2.30.0) and extended by 660 bp each side using bedtools slop. Each replicate was normalized per million reads mapped in their own peaks, and the displayed coverage is the mean of normalized replicates.

### ChIP-seq

For genomics analyses, we used the mouse assembly mm10 and the chicken galGal6 and plotted genomic data using pyGenomeTracks 3.6 (Lopez-Delisle et al., 2021). ChIP-seq experiments were performed using the ChIP-IT High Sensitivity Kit according to the manufacturer's instructions with minor modifications (Active Motif). For the mouse samples, 12 pairs of E12.5 whisker pads (WPs) or 100 E8.75 posterior trunks were used, the latter dissected at the level of the second to fourth pair of somites. For the chicken samples, the dorsal skin of five HH35 embryos, the posterior trunk or 80 HH19-21 posterior trunk regions were isolated. In all cases, the samples were fixed for 10 min in 1% formaldehyde at room temperature and the crosslinking reaction was quenched with glycine. Subsequently, nuclei were extracted and sonicated in 50 mM Tris-HCl (pH 8.0), 10 mM EDTA and 1% SDS to an average fragment size of 200-500 bp using a Bioruptor sonicator (Diagenode). Approximately 10-20 µg of sonicated chromatin was diluted tenfold with the dilution buffer [20 mM HEPES (pH 7.3), 1 mM EDTA, 150 mM NaCl, 0.1% NP-40] and incubated with 2 µg of anti-H3K27ac antibody (Abcam, ab4729). All buffers contained 1× Complete Proteinase Inhibitor cocktail (EDTA-free; Roche) and 10 mM sodium butyrate to prevent protein degradation and histone deacetylation. Chromatin–antibody complexes were immunoprecipitated with protein G-coupled agarose beads (Active Motif), purified and de-crosslinked according to the manufacturer instructions. Approximately 5-10 ng of purified DNA was used for ChIP library preparation by the Geneva IGE3 Genomics Platform (University of Geneva) and sequenced to obtain 100 bp single-end reads on an Illumina HiSeq2500 or HiSeq4000 system. Published fastq files were downloaded from the Sequence Read Archive (SRA) for H3K27ac: mouse distal forelimb (DFL; GSM2713703, SRR5855214), mouse proximal forelimb (PFL; GSM2713704, SRR5855215), chicken DFL (GSM3182462, SRR7288104), chicken PFL (GSM3182459, SRR7288101); and for CTCF in mouse posterior trunk (PT; GSM5501395, SRR15338248). ChIP-seq reads processing was done as in Amândio et al. (2020) with some modifications, using the laboratory Galaxy server (Afgan et al., 2016). Adapters and bad-quality bases were removed with Cutadapt (v1.16.8) (Martin, 2011) (options -m 15 -q 30 -a GATCGGAAGAGCACACGTCTGAACTCCAGTCAC). Reads were mapped to the mouse genome (mm10) and to the chicken genome (galGla6) using Bowtie2 (v2.4.2) (Langmead and Salzberg, 2012) with standard settings. Only alignments with a mapping quality >30 were kept (Samtools v1.13) (Danecek et al., 2021). H3K27ac ChIP-mapped reads were downsampled with Samtools view (v1.13) according to the sample with the smallest number of reads after filtering and duplicate removal (mouse: 71,741,225 reads; chicken 25,872,853 reads). Mouse CTCF ChIP-mapped reads were downsampled to a quarter and chicken CTCF ChIP reads were not downsampled. When two replicates were available, alignment BAM files were merged using Samtools merge (v1.13) and subsampled to the same number as single replicates. The coverage and peaks were obtained as the output of MACS2 (v2.1.1.20160309.6) (Zhang et al., 2008) with a fixed extension of 200 bp (--call-summits --nomodel --extsize 200 -B) and, for the WP sample, the q-value cutoff for peak detection was set to 0.1 instead of the 0.05 default. CTCF motif orientation was assessed using peaks extended by 100 bp each side and the CTCFBSDB 2.0 database (Ziebarth et al., 2012) with MIT_LM7-identified motifs. Open H3K27ac peaks were obtained by the intersection of H3K27ac peaks with ATAC peak regions in matching tissues using betdtools intersect. The proportions of open H3K27ac peaks were obtained using R (v3.6.1) and the bar plots of Fig. S5A were made with the ggplot2 package (v3.2.1) (https://www.R-project.org/).

### MAR annotation

MARs were defined as genomic segments of the T-DOM with the highest density of specific H3K27ac coverage and were annotated as follows: for mm10, chr2:74,872,605-75,696,338 and for galGal6, chr7:15,856,252-16,252,401; sliding windows were of 10 kb every 2 kb. For each window, the coverage of the ChIP was computed and the difference with the coverage in the corresponding H3K27ac ChIP in the brain was considered as the specific signal. The smallest sequence of consecutive windows cumulating 30% (for the dorsal skin and WP samples) or 50% (for the PT samples) of the total specific signal is the MAR. See ChIP_scanRegionForHighestDensity_2files.py at https://gitlab.unige.ch/Aurelie.Hintermann/hintermannetal2022 for details.

### Annotation of open pCREs and pCREs

pCREs were defined as non-coding genomic intervals that overlap with H3K27ac MACS2 peaks in at least one of the samples analysed per species. To obtain the genomic coordinates of pCREs, MACS2 peaks of all samples were first pooled into one file, the size of each peak was increased by 660 bp each side to account for nucleosome occupancy using bedtools slop (v2.30.0) and overlapping MACS2 peaks were combined into a single interval using bedtools merge. Intervals were annotated according to their colocalization with tissue-specific H3K27ac peaks, exons, promoters and sequence conservation using bedtools intersect. Intervals overlapping with exons were discarded to obtain pCREs, which were then further annotated as ‘enhancers’ and ‘promoters’ depending on whether they mapped more or less than 2 kb away from a gene TSS, respectively. pCREs that were H3K27ac positive in more than one tissue were called ‘pleiotropic’, whereas those being active in one tissue were labelled ‘specific’. Open pCREs are H3K27ac peaks which overlapped with ATAC peaks using bedtools intersect. pCREs were quantified using R (v3.6.1) and the bar plots of Fig. 5A were made with the ggplot2 package (v3.2.1). See CNEs_01.sh at https://gitlab.unige.ch/Aurelie.Hintermann/hintermannetal2022 for details.

### CNEs

Pairwise BLASTZ alignments were downloaded from http://hgdownload.soe.ucsc.edu/goldenPath/mm10/vsGalGal6/mm10.galGal6.synNet.maf.gz and transformed to intervals with maf to interval (Blankenberg et al., 2011). Conserved elements were considered to be non-coding if they did not overlap with annotated exons either in mouse or in chicken. The synteny plot on Fig. S3A was made using the R package ggplot2 (v3.2.1). H3K27ac peaks and open H3K27ac peaks were first extended by 660 bp on each side to account for nucleosome occupancy. Then, CNEs that were located 2 kb or more from the next TSS and which intersected with either H3K27ac peaks or with open H3K27ac peaks using bedtools intersect were annotated as enhancer CNEs and open enhancer CNEs, respectively. Enhancer-CNE heatmaps were made with the pheatmap R package (v1.0.12) using a matrix in which columns were enhancer CNEs (Fig. 5B) or open enhancer CNEs (Fig. S5B), rows were the tissues in which acetylation peaks were observed and values were the scores of acetylation peaks. If an enhancer CNE comprised several peaks of the same tissue, the average was calculated. In Fig. 5C and Fig. S5C, the relationships of the three sets formed by the conditions of being an enhancer CNE (or an open enhancer CNE) in the mouse tissue, in the chicken tissue or in a given mouse tissue, in the matching chicken tissue or in another chicken tissue were represented on Euler diagrams using the eulerr R package (v6.0.0), in which each tissue analysed was considered independently. See CNEs_01.sh at https://gitlab.unige.ch/Aurelie.Hintermann/hintermannetal2022 for details.

### 4C-seq experiments

4C-seq experiments were performed according to Noordermeer et al. (2011). Briefly, the tissues were dissected, dissociated with collagenase (Sigma-Aldrich/Fluka) and filtered through a 35 μm mesh. Cells were fixed with 2% formaldehyde (in PBS with 10% fetal bovine serum) for 10 min at room temperature and the reaction was quenched on ice with glycine. Nuclei were extracted using a cell lysis buffer [10 mM Tris-HCl (pH 7.5), 10 mM NaCl, 5 mM MgCl_2_, 0.1 mM EDTA] and stored at −80°C. Approximately 200 E9.5 posterior trunk regions dissected at the level of the second to fourth pair of somites, as well as 120 chicken HH19-21 posterior trunk samples, were used. Nuclei were digested with NlaIII (New England BioLabs) and ligated with T4 DNA ligase HC (Promega) in diluted conditions to promote intramolecular ligation. Samples were digested again with DpnII (New England BioLabs) and re-ligated with T4 DNA ligase HC. These templates were amplified using the Expand Long Template (Roche) and PCR-barcoded primers flanked with adaptors. For each library, eight to ten independent PCR reactions were pooled together and purified using the PCR purification kit (QIAGEN). Multiplexed libraries were sequenced on Illumina HiSeq 2500 to obtain 100 bp single-end reads. Demultiplexing, mapping and 4C-seq analysis were performed using a local version of the pipeline described in David et al. (2014) on the mouse mm10 and chicken galGal6 genome assemblies. The profiles were smoothened using a window size of 11 fragments and normalized to the mean score in ±5 Mb around the viewpoint. When multiple independent biological replicates were available, average 4C-seq profiles were calculated. When viewpoints were subtracted, the score of each window of the normalized, smoothed profiles were subtracted (see 4C_subset_subtract at https://gitlab.unige.ch/Aurelie.Hintermann/hintermannetal2022) for details.

### Annotation of the D-regions

The definition of the interacting regions was based on 4C-seq interaction profiles in mouse and sequence conservation. The regions are delimited by CNEs to be able to transpose them to the chicken genome. CNE331 was chosen for the 5′ border of the D1-region as it is the only CNE located in the region that spans from the 3′ of the *HoxD* cluster to the promoter of *Mtx2*. At the other extremity, the 3′ border of the D9-region was set to CNE382, where the *Hoxd9* signal goes back to the same level as *Hoxd4* (Fig. S3A). The limits between the *Hoxd1*- and D4-regions and between the D4- and D9-regions were established based on 4C scores normalized by the number of mapped reads. To obtain the relative distribution of contacts across our region of interest (i.e. from CNE331 to CNE382), we first normalized 4C scores by the total score of the whole region. We then computed the cumulative sum of the difference between the normalized scores of two viewpoints (Fig. S3C). Accordingly, the delimitation between *Hoxd1*-region and D4-region was set to CNE346, which is the closest conserved element to the minimum value of the cumulative sum using the score difference between *Hoxd4* and *Hoxd1*, and thus corresponds to the end of the genomic segment where *Hoxd1* values are higher than those of *Hoxd4*. Similarly, the D4/D9 border was set to CNE364, which corresponds to the genomic location for which the cumulative sum of the score difference between *Hoxd9* and *Hoxd4* is minimum and where *Hoxd9* values become higher than those of *Hoxd4*. The limits of the D-regions were then transposed to the chicken genome using the corresponding CNEs. Finally, the normalized 4C scores for each viewpoint were quantified in each region and normalized by the region size in Mb (Fig. S3D). The D-regions were quantified using R (v3.6.1) and the bar plots of Fig. S3D were made with the ggplot2 package (v3.2.1).

### Ethics approval

All experiments involving animals were performed in agreement with the Swiss Law on Animal Protection (LPA), under licence no. GE 81/14 (to D.D.).

## Supplementary Material

Supplementary information

Reviewer comments
