# Supplementary material for: Developmental and evolutionary comparative analysis of a regulatory landscape in mouse and chicken
Source: Development. 2022 Jun 30;149(12):dev200594. doi: 10.1242/dev.200594 (PMC9307994; doi:10.1242/dev.200594)
Supplement: Supplementary information [file develop-149-200594-s1.pdf]

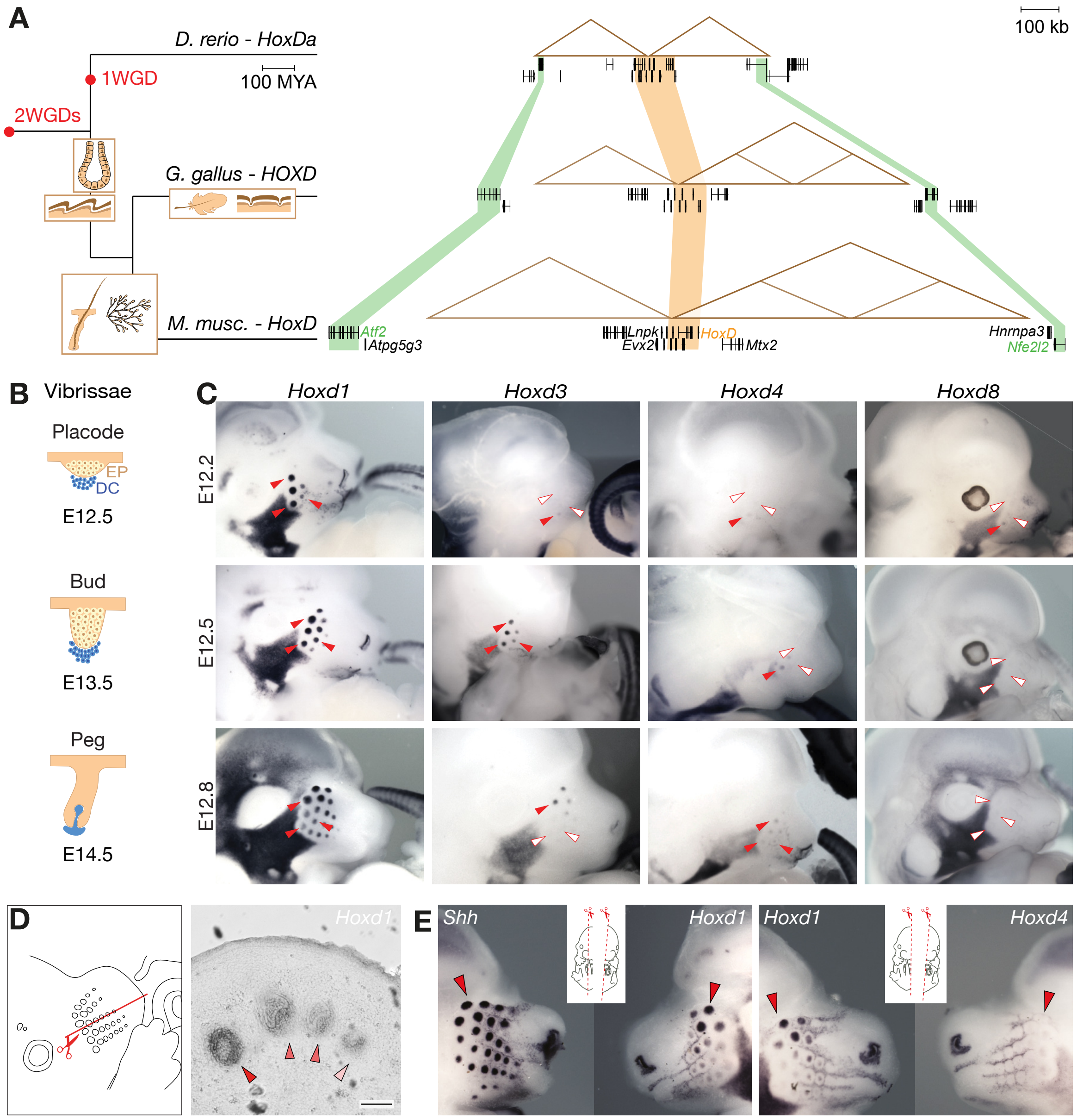

Fig. S1. Evolution of the *HoxD* locus and expression of *Hoxd* genes in the developing VPs. A. On the left is a schematic illustration of a vertebrate phylogenetic tree showing the emergence of some skin derivatives (glands, scales, feathers, hairs). On the right are the corresponding versions of the *HoxD* locus, with the orthologo us *HoxD* gene clusters as shaded orange areas and other orthologous genes shown in green to help delimit the ext ent of the conserved regulatory landscapes. The commonly accepted position of whole genome duplication (WGD) events in the phylogenetic tree are represented by red dots; 2WGDs refers to the two events that occurred at the base of the vertebrate lineage whereas 1WGD refers to the additional duplication event that occurred at the base of the te leost lineage. **B.** Schematic representation of vibrissae morphogenesis: epithelial placode (EP, yellow cells); dermal condensation (DC, blue cells). The approximate developmental stage is indicated below, showing the delay between vibrissae and hair morphogenesis. The dorsal skin of the upper back was used as reference for hairs. **C.** WISH on th ree successive E12 staged embryos (indicated on the left) using four *Hoxd* genes as probes (top). The same three pla codes are pointed by red (positive) or white (negative) arrowheads. **D.** Schematic of the transversal section on E13.5 VPs (left), analyzed in ISH with a *Hoxd1* probe (right). The scale bar in the right picture corresponds to 100 µm an d the *Hoxd1* stained VPs at peg stage (see below) are indicated by red arrowheads of decreasing intensities from lef t to right. The staining is localized in dermal cells. **E.** WISH on E12.5 bisected mouse embryos. *Hoxd1* mRNAs were detected after those for *Shh* (left) and before *Hoxd4* mRNAs (right).

Development • Supplementary information

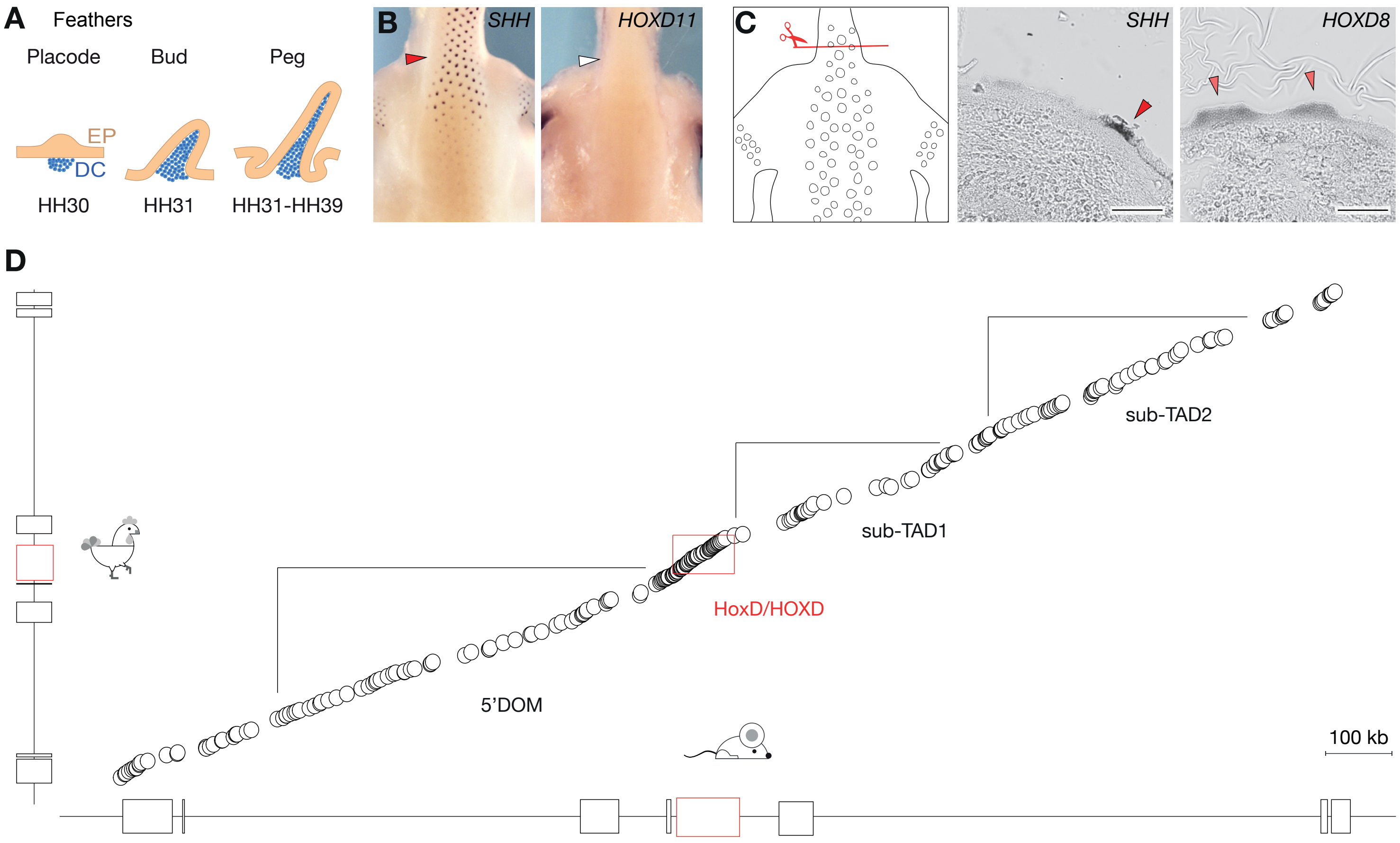

Fig. S2. Feather development and transcription of *Hoxd* genes. A. Schematic of feather development between stage HH20 and HH35 with ectodermal placode (EP, yellow) and dermal condensation cells (DC, blue). **B.** WISH on HH35 chicken embryos, dorsal view of the upper back, with arrowheads pointing toward t he neck at the shoulder level. *HOXD11* transcripts were not detected in FPs (right), unlike those of SHH (left). **C**. Transversal section through a HH35 chicken skin of the upper back (red), corresponding to the two sections staine d by ISH on the right. The scale bar is 100 µm. *SHH* is used as marker of the epithelial placode (red arrowhead). *HOXD8* is also transcribed in epithelial placodes, matching -or slightly larger than- the *SHH* domain. **D.** Synten y plots representing sequences conserved between the mouse and the chicken *HoxD* loci. On the X-axis is the mou se locus (mm10, chr2:73800000-75800000) and on the Y axis is the chicken locus (galGal6, chr7:15790000-16700 000, inverted x-axis). The scale is the same for both axes. TADs and sub-TADs are indicated by black lines and the *HoxD* cluster by a red rectangle. Schematic representations of the locus are on top of each axis. Despite a mouse l ocus that is in average 2.2 times larger than its chicken counterpart, the order of the conserved sequences is maint ained, showing the absence of substantial genomic rearrangement at this locus.

Development • Supplementary information

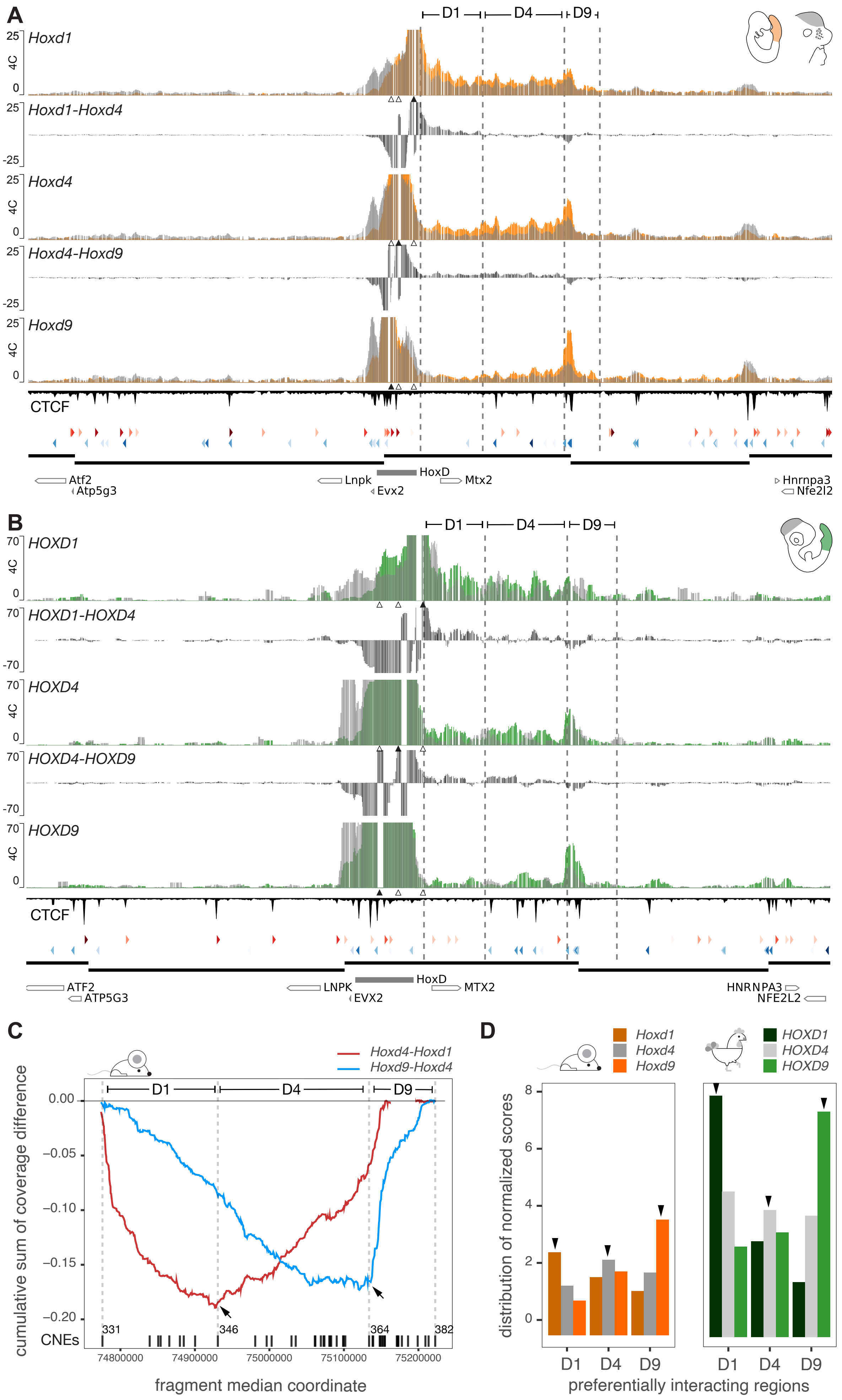
A
D1
D4
D9
Hoxd1
Hoxd1-Hoxd4
Hoxd4
Hoxd4-Hoxd9
Hoxd9
4C
CTCF
HoxD
Atf2
Atp5g3
Lnpk
Evx2
Mtx2
Hnrnpa3
Nfe2l2
B
HOXD1
HOXD1-HOXD4
HOXD4
HOXD4-HOXD9
HOXD9
ATF2
ATP5G3
LNPK
EVX2
MTX2
HNRNPA3
NFE2L2
C
Hoxd4-Hoxd1
Hoxd9-Hoxd4
cumulative sum of coverage difference
CNEs
fragment median coordinate
D
distribution of normalized scores
preferentially interacting regions
HOXD1
HOXD4
HOXD9

Fig. S3. Comparative 4C-seq analyses at the mouse and chicken *HoxD* loci. A. On the Y axis are 4C-seq normalized scores per feature, with E9.5 mouse posterior trunk cells scores (orange) superimposed over E12.5 FB cells (grey). Below the 4C tracks, the positions of the *Hoxd1, Hoxd4* and *Hoxd9* viewpoints are represented by triangles, with a black triangle for the viewpoint used in the track. Between each track, the subtraction of the two adjacent viewpoints is represented (black). The preferentially interacting regions D1, D4 and D9 were annotated manually and, to facilitate comparisons between their positions in the mouse and the chicken locus, orthologous CNEs located close to the edge of each region D are shown (vertical dashed lines). CTCF ChIP-seq profiles are displayed below the 4C tracks, with red and blue arrowheads below indicating their orientation. Color intensity is proportional to motif score. Data mapped on mm10, chr2:73800000-75800000. **B.** On the Y axis are 4C-seq normalized scores per feature, using HH18 chicken posterior trunk cells (green), superimposed with the profile obtained with HH18 brain cells (grey). Data shown for *HOXD1* (top) *HOXD4* (middle) and *HOXD9* (bottom) viewpoints, with a black arrowhead indicating the position of the viewpoint. Reads mapped on galGal6, chr7:15790000-16700000, inverted x-axis. **C.** On the Y axis is the cumulative sum of the difference between normalized scores of two adjacent mouse 4C viewpoints for each genomic fragment; the median coordinate of the fragments is shown on the X axis. The minima are indicated by black arrows and represent the genomic location where preferential interactions switch from one viewpoint to the next; from *Hoxd1* to *Hoxd4* (red line) and from *Hoxd4* to *Hoxd9* (blue line). **D.** Barplots showing the relative distribution of the 4C normalized scores throughout preferentially interacting regions, for each viewpoint. Mouse quantifications are in orange, chicken in green. Black arrowheads indicate the viewpoint which has the higher score, in each region. Each viewpoint's score is higher with its associated region than with the other regions (i.e., *Hoxd1* interacts more with the D1 region than with the D4 and D9 regions). Moreover, each viewpoint's score is higher within its associated region than the score of other viewpoints (i.e., *Hoxd1* interacts more with the D1 region than *Hoxd4* and *Hoxd9*).

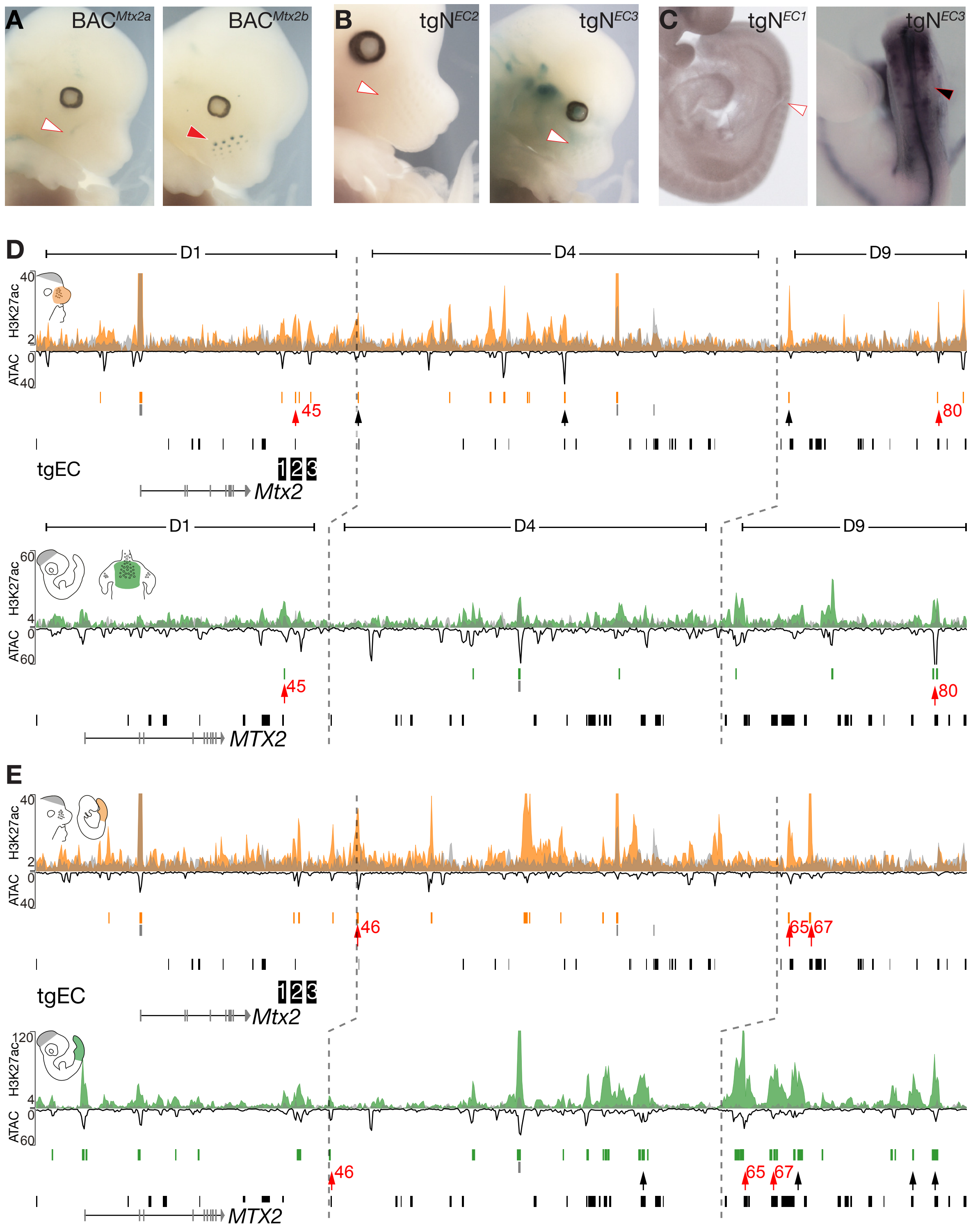
A
BACMtx2a
BACMtx2b
B
tgNEC2
tgNEC3
C
tgNEC1
tgNEC3
D
D1
D4
D9
H3K27ac
ATAC
45
80
tgEC
Mtx2
MTX2
E
46
65
67

Fig. S4. Comparison of conserved and non-conserved CREs in mouse and chicken embryonic tissues. **A.** X-gal staining of E12.5 transgenic embryos carrying either the first half (left) or the second half (right) of BAC^{Mtx2}. The entire BAC^{Mtx2} recapitulated the pattern of expression of *Hoxd1* in VPs. **B.** Staining of embryos carrying either the TgN^{EC2} or the TgN^{EC3} transgenes, which show no regulatory activity in VPs at E12.5. **C.** Staining of E9.5 mouse embryos carrying the TgN^{EC1} or the TgN^{EC3} reporter constructs, which show the absence of signal in forming somites. **D.** H3K27ac ChIP-seq and ATAC-seq profiles magnifications over the mouse and chicken sub-TAD1. Top: Mouse E12.5 dissected VPs (orange) along with the mouse E12.5 forebrain cells track (grey)(mm10, chr2:74775737-75222876). Below is the profile for the chicken HH35 dorsal skin (green), superimposed to HH18 brain cells (grey) (galGal6, chr7:16033612-16252401, inverted x-axis). ATAC-seq profiles using E12.5 VPs and HH35 FPs are shown as a black line with inverted y-axis below the H3K27ac coverage to indicate the levels of chromatin accessibility. Open pCREs (H3K27ac, MACS2 narrowPeaks, which overlap ATAC peaks) are in orange/green vertical lines and blastz conserved sequences in black vertical lines, below each track. The peaks overlapping CNEs are pointed by black arrows. The peaks corresponding to CNEs in both species and in equivalent tissues are pointed by red arrows. Vertical dashed lines represent the position of corresponding CNEs at the edge of each region **E.** Open pCREs focusing on sub-TAD1. Top: The profile for mouse E9.5 posterior trunk cells (orange) are superimposed to mouse E12.5 forebrain cells (grey). Bottom: Profiles of chicken HH18 embryonic posterior trunk cells (green) and of HH18 brain cells.

Development • Supplementary information

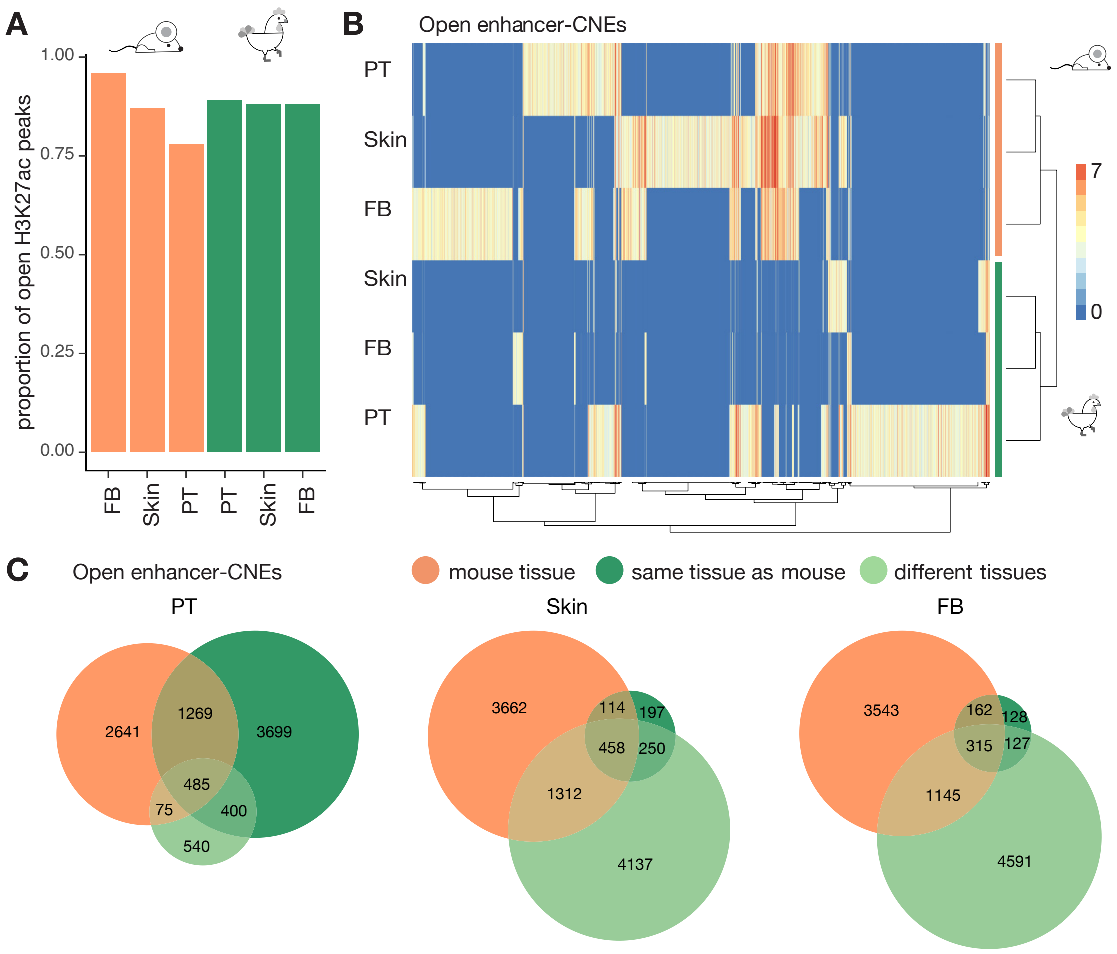

Fig. S5. Verification of the H3K27ac based approach with ATAC-seq data. A. Bar plots showing the proporti on of 'open H3K27ac peaks' (i.e., H3K27ac peaks which overlap with ATAC-seq) in mouse (orange) and chicke n (green) tissues. A large majority of H3K27ac peaks have an ATAC-seq peak in the matching tissue. **B.** Hierar chical clustering obtained using the pheatmap R package. On the *X*-axis are shown open enhancer-CNEs i.e., con served sequences, which are non-coding in mouse and in chicken and which overlap with both a H3K27ac and an ATAC-seq peak in at least one species. On the *Y*-axis are displayed those tissues used to obtain MACS2 process ed peaks. The values on the heatmap correspond to the enrichment scores of the peaks. Open enhancer-CNEs clu ster by species and not by tissues, similar to when enhancer-CNEs are used without accounting for chromatin acc essibility. **C.** Euler diagrams representing the proportion of open enhancer-CNEs, per tissue, active in mouse (ora nge), in the corresponding chicken tissue (dark green), or in unrelated chicken tissue (light green). The proportion of open enhancer-CNEs diverging in their regulatory activities is comparable when the regulatory function is approximated by the presence of both H3K27ac and chromatin accessibility status or solely by H3K27ac as in Fig. 5C. In **B.** and **C.** PFL, proximal forelimb; DFL, distal forelim b; PT, posterior trunk.

Table S1. List of mouse lines.

Line	Reference	Coordinates	Primers for genotyping
Del(1-13)d9lac	(Rodriguez-Carballo et al., 2017)	chr2:74663889-74764314	F : GACCGCTTATCCAATTCGGTTC<br>R : CAAGGTCCTCAGCCTTAAGAGTGG
Del(Attp-SB2)	(Andrey et al., 2013)	chr2:74765140-74916570	F: ATCCCGGGGGATCCACTAGAG<br>R: GCTATATAGTAGTTT CAAGGCCAGCCC
Del(Attp-SB3)	(Andrey et al., 2013)	Chr2:74765140-75767350	F: ATCCCGGGGGATCCACTAGAG<br>R: GCTTCCCTAGTTTATGGGGGAGG
Del(SB2-SB3)	(Schep et al., 2016)	chr2:74916560-75767350	F: ATCCCGGGGGATCCACTAGAG<br>R: GCTTCCCTAGTTTATGGGGGAGG
TgBAC^{Mtx2}	(Allais-Bonnet et al., 2021)	chr2:74747769-74913171	
TgBAC^{T1}	BAC1 in (Delpretti et al., 2013)	chr2:74918538-75112340	F: CCTGCTGATGAAGCAGAACA<br>R: CAGCGACCAGATGATCACAC
TgBAC^{T2}	BAC7 in (Delpretti et al., 2013)	chr2:75171805-75354437	
TgBAC^{HoxD}	(Schep et al., 2016)	chr2:74563168-74777972	F: GGTAAACTGGCTCGGATTAGGG<br>R: CTATTCAAAGGTGGGGAGCAGTC

Development • Supplementary information

Table S2. Primers used to clone enhancer candidates.

CRE name	Coordinates	Primers for cloning
WPAE1	chr2:74780382-74782582	F : GCTGCAGGAATTCGATATCAGAGTAGG<br>R: CGAGGTCGACGGTATCGATAGAATAGA
TPAE	chr2:74809810-74811023	F : AGGGCCATTGTCCTCCATTCTT<br>R : CGTGTGTGTGTGTGTGTGAAGG
WPAE2	chr2:74892377-74895558	F : TCCTCTCAAATTTTCCTAACCCCCA<br>R : CCCACCTGCCTCTACATAGCTC
WPAE3	chr2:74898212-74903231	F : CTGTCACCACCCCAGTCCTG<br>R : AAAAAGGGGGAGTGGGTGGG
WPAE4	chr2:74905913-74910158	F : AGGTTCCTGTCTTCGTGTTGG<br>R : GTAGCTCACCAGGGCTGAGG

Development • Supplementary information

Table S3. Probes for *in situ hybridization*

Mouse

Probe name	Reference	Digestion/ Transcription	Sequence
Hoxd1	Lab (AH)	NcoI / Sp6	GTCAAAATCTTTACATGCTATGCATGACATTAAATGTCATATATAAGGTTCACTGCAAGCAAATCTTTAAAAAATATACATACTGTATATATTTACACTTCAAGTTTGAGTAACTGATGCCAA<br>GGTATAATTCAGATTCTAATCCTTACCTACCCTGTGGATTTGTTCCGCTACTAACTAAACAGTGATTGTGTTTCTTCACCCAGGCACACGAAGAGGTAGGAGCGCCACTTTAAATAAACAAAC<br>TGGAGCACAATATGAAGATCATCATTGAAGCAGCTTAGCTACATAGAGGAAACCCTAGCTAGCTTTGATCATCTCAGGAAGCTGAATATATGTAGTCTGTCCAGGAAATTAAAACTGAAAC<br>AACTTCAGAGAGATACGTTTCTGACCACACTAGACTAAGAAGGCCTGCCACTCTGCACCCGAGAATTTTAAGTGGCTCACATAGCAGCGTTTAGGCAGGTAGCTAAGACACCTTTCCTCCCC<br>ATGAGGCAAAAATAATATGCGAATAAAAGATATCAAATATATATCTCTATACACAGCCTCTGGAACTCAGAAGCCAATTTAGACAATTCCTTTGCCAAGTAAAATCCGTATTAATTTCTACC<br>CATGCCCACCTCCAGCCCAAACTAAGCTCTCAAGTCTATTCAAAGGTGGGGAGCAGTCTGTGACTTCTGTAGACCCAA
Hoxd3	(Condie and Capecchi, 1993)	PmlI / T3	CTACAAATATACACATAAATACATTAAGACGAGGATAAATAATCAGGGAAACGAAATTACTCGCTTCAAATAATAAATACAGATTGTTTAGTCCGATGCAATCTTTACAGGAGGGGACGGA<br>GCATTATAAATTAAGGTCTCTGAAACTAAGCTAGACAGAAGGGCCAGGCCCAGGGTTCTCCAGGCCCTAGTCCCTCTTCAGGACTCGCCACGGATGGGTCGAGGACTTACCTTAGGGACACC<br>ATTACCAGATTAGAGTCCCTCGGTCCGCAGTTTATGGCACTGTCAGGTTTACTGGGGAGGATCCTTGCGGCTGATTTATGGCGAGTCACCCAAAGGAGGACGCGCCTGGAGGGAGGCCCAGT<br>CGCGTCAGCCCTGGCCGCTGGAAGCCACTCGTGAGGCATCCGCCAGGCCAGATCAGGGAGGGGGATCCCCTAAACTGCCCCTCGGGCCCCGCCCCGCCCCACCACCACCTCTGCCACCAAA<br>ACCAGAGAGGTAATTGCGCCTGGCCGGCGGCAACAGCTACAGATGTGTCAGTTTGGGGGCCTCGGGCAGGCGTCCCTGAGACGAGTGGTGAGCCGAGAGATCTGTGTAGGTGGGATGAGG<br>GTCGCAGGGTCCGTGGTGATGGTTGCCAGGGATTTGCGCGGCGCAACTGTAGTCCACGCTGGCCGAAGACGGGTGCGAGAGGTGGCCCAGATTGAAGACCGGCCCGGACGTGGGCGCCATG<br>GAGTCGACGAAGTTGCCGCCCACGTACACCGGGCTGCCCTGCAGGTTGGCGCTGGCGAAGCCGCCGCCGTTGCTCGCCATGGGGTGAGGCTCGAACTCGGGCGCCGGGTACCGCTTCTGCT<br>GAGGCAGGCAGCTGCTGAGCGGCGCCGTGTAGGCGGCCAGGCCGTACATATTGGGCTGCGATTTGGCGAAAGCGGGCGGCGAGGGTGCGTCGTAGGCCAGGCCGGGCACCGGCGGCAGCT<br>GGCCGGAGTAAGCCAC
Hoxd4	(Featherstone et al., 1988)	EcoRI / Sp6	TAAGACTATTCACCATCTTAAGGTTTTTGGCACGCAGGTGTCGGTTATTTTCTGCAGTCCATTCCCCGAACAAATAAATATTTAAGCAAATATAAACTAAACATAAAATAACCCGTTTCCTTC<br>TTGGATTTATTTCTTTGGGCATGGGCCACTGGAAGCAAACAATTTCTTTCTTTCTTTTTTTTTTTTTTTTTAAATCACAGAAGAAATTCTATAGATAATCCAGAAAGTTCTGGGATTCTGGCTCAG<br>CCTCTCCTGCTTGCACTGAAAGCACCTAGCACTGGCTGGCCTTCTGCCTCAACTCCCCCACAGTTACTAAGCCACACAGCTCCCTGAAGGGGGTGGTGTGGAGGAGACCATCCCGGTTCCCA<br>CTGGGGTAAAGCCTCCAACCTTCATTTGCAAATTCAATCCACCAGCAGAGATTTTCAGGAGTTTAATGACTCGCCAAGGGTTTATGTTCTCAGTAGCTCCTGCCATATGATAATCTACTTAAA<br>GAAGCCCGCCCACCCTCCCACCCTTCCCCACTTTAGGGAGGGGGCTGCCCTCGGGCAGGAAGGTAACCTAGTCCGAGGGCGGCCTGCAGGTCCCAGGTCCACCCCCACAGTACCTTAGAGC<br>CCAGTGAGGTGACAGCAGGCCCCGCCTGCACCCGCAGCTTCGCCTCAACCTGGAGTGCAAGAAGGGATAGGCCCAGGGTCCCCACTTCTATAAGGTCGTCAGGTCCGTATGGTGGTCCTTGG<br>CCATTGGCTGCAAATGCTGGCCCGGGGCAGCTGAAGAGGAGCAGGAAGATGAGGAAGAAGACCTGCCCTTGGTGTTGGGCAGTTTGTGGTCTTTTTTCCACTTCATCCTCCGGTTCTGGAAC<br>CAGATCCCCGGGCGAGCTCGAATTC
Hoxd8	Lab (JZ)	EcoRI / T7	TGCAGGCATAATTTTTAAAAACATTATTAATAAACTTCTTTTAATTTACTCACCATAAAGATTTAAAATGTTCAAGTGCACTTGTTTGATTAATAAAGCATTTTATATTCAATCATCTAGCGCT<br>GATCATTAGGAAGTTTATGTTGTTGAGGCAAACCACCAGCTCAGACGTTAAAAATTACCATTATAAAAATATAGCCACAAGGTCCGTATCAAATTACCCTTCTATAGGACAGCTCAAGGACT<br>GCTTCTATGCGATACTACAAACTATTCTCTTTAAAAACACAGAAATCCCTTTCAAAGCGCAGAAACCTCAAGAGCAGTGACATTAAGGTAGCTTGAAACACAGGTAAAGTCCGTTTCCAAAA<br>CACAAGAGGGAGGACCGGTGGTGTCCACCAGTATATACTTATACTGATCATCAGTCTTAATAGTCTGTGGTAGAAGTGTAAAGGTAGAGTTTAATTTGTGGGGCAGCCTTCAGCTCCGTCTT<br>CTTCCAGCCCCGAGACTTCCTTTTTGGGGTCTCCATCCTTTGCCTCCGGCCGGGACGCAGGAAACTTGTCTTTGTTGTTCTCCTTTTTCCATTTCATTCTCCTGTTCTGGAACCAGATTTTTACCT<br>GTCTCTCCGTGAGGGCCAGAGTATGGGAGACCTCGATTCTCCTCTTCCTGGTCAGATAAGGGTTAAAAAGGAATTC
Hoxd9	(Dollé et al., 1989)	BamHI / T7	CTGAGTTGGATTTTAGTTGGGAGAGCAGGGTGGGGGTGTCCACACTCTGTCTTCCTTCAGATTCTCGGAATTAGATCGTTGGCCCTGTTTGGGAGGCTGGATAGGGTGAGACTAAACTACAG<br>CACTTTTCAGAAACATGAGGGACATTTACATAGAATAGGGCTCCATCAAGCCTGCGTGCACTCACAAAATATAGCATTTCCTAAGGAATCAGGGGAGCAGGGAAGGGAGGGAGTGGGACA<br>GGACAAGACTCCAAAGCCACTTGGTTTCTCCAATACATATGCGAGCAAATACAGTGTCCCCCAGATAACTCACAGAGACCAACCACACAGTCACTTCTACCTAAAAAAAGAAGAAAGTCCG<br>AGTCGCTGCAGAGTTTCTGAATCAAGCACCCACAAAGAAAACAAACAACAACAAAAAACAAAAAACAAAACAAACAAACAAACAAACAAAAAAACCCGCTGCCTTTTGAGTGCTGCAGGC<br>CAGACCCGCACTGGGTCAGTCTCCTTTAGGGCACTTCTCCTTGCTCATCTTTTTCATTTTCATCCTACGGTTCTGGAACCAGATTTTGACTTGTCTCTCTGTAAGGTTCAGAATCCTGGCCACCT<br>CGTAGCGCCGGTCCCGGGTGAGGTACATGTTGAAGAGGAATTCCTTCTCCAGCTCTAGCGTCTGGTATTTGGTGTAGGGACAGCGCTTTTTCCGGGTGGAGCGAGCGTGGATC
Shh	(Echelard et al., 1993)	HindIII / T7	GCTGACCCCTTTAGCCTACAAGCAGTTTATTCCCAACGTAGCCGAGAAGACCCTAGGGGCCAGCGGCAGATATGAAGGGAAGATCACAAGAAACTCCGAACGATTTAAGGAACTCACCCCC<br>AATTACAACCCCGACATCATATTTAAGGATGAGGAAAACACGGGAGCAGACCGGCTGATGACTCAGAGGTGCAAAGACAAGTTAAATGCCTTGGCCATCTCTGTGATGAACCAGTGGCCTG<br>GAGTGAgGCTGCGAGTGACCGAGGGCTGGGATGAGGACGGCCATCATTCAGAGGAGTCTCTACACTATGAGGGTCGAGCAGTGGACATCACCACGTCCGACCGGGACCGCAGCAAGTACG<br>GCATGCTGGCTCGCCTGGCTGTGGAAGCAGGTTTCGACTGGGTCTACTATGAATCCAAAGCTCACATCCACTGTTCTGTGAAAGCAGAGAACTCCGTGGCGGCCAAATCCGGCGGCTGTTTC<br>CCGGGATCCGCCACCGTGCACCTGGAGCAGGGCGGCACCAAGCTGGTGAAGGACTTACGTCCCGGAGACCGCGTGCTGGCGGCTGACGACCAGGGCCGGCTGCTGTACAGCGACTTCCTCA<br>CCTTCCTGGACCGCGACGAAnGCGCCAAGAAGGTCTTC

Development • Supplementary information

Chicken

Probe name	Reference	Digestion/ Transcription	
HOXD1	Lab (AH)	SalI / T7	GAAAGCTTGTTTGGGGTCAGCGCTTTAACAAGGAACTGCCCACCCCTTATTTTGGATGTTTGTATTTTCGTACAAATTTCATATAAGCAGCTCCACCCTGGGAATTTATGTTGTTTCGTTTT<br>TTAACTCGTGAAGTGCATCAGCTCTTTGGAAGTGAGCTGTAACACTGTGCACGAAACCTgAAAGTCTCTGTGTGCgAGCAAGGCCATGAGCCTCTGCCCAGGGTATGGTGTGTGACTATGG<br>TGTGTGACTAGATCTCAGCAGCTGTTGGTAGCAGGGGCTGCTTTCCAGGTGTGGATCCACGCTTCCAGGTCCTGGAGATCCCTCTCCCAgTGCTATGTTTTTCTTTCaCCTGAGTGAAATCC<br>AAAGCAAACTGCCAGCTCTTTCaTCCCCTAAATCCGGGTGGCCTCCCTATCTGTGCTGCCATTTATACATTCCCAGTCATCAGTCAGGTCCAGAAAAACCTCTGAATCCAGCTTCTCTGTCC<br>ACTTTGATACCATTGTTTTACCTCTGGAAAAGATGGAATCTGGGGAAGTGAGGATTGCTGGTGAGTGTTGCACTTCAGTGGAGTAGCTGGGGTTGTCACTGCTTTAGAACTGGTTCTCTGG<br>GAGGGGAGAGACCCAGGAACTCGCATGGTCTCACTGACAGAGGTCTGCAGTCAAACCCAAACATCTTTATGCTCTTTAACAGCTCTTAGCAAACTGGCTTAATATGGTACTTTTCTTTAA<br>ACATTAGATACAGTAAAGCCTAgGAGAGcCCCCGTGCTATGAGAGGCTCTACACTGTCCTTGCCCACACACTGGGAGGCAGaGGAGTGGGCACTGGCCCTGCAGaGGGCACGCaGGGCAG<br>CCTTTCTCCTGATGGACTGTTTGTTC
HOXD3	Lab (AH)	SalI / T7	TAATACGACTCACTATAGGGCGAATTGGGCCCGACGTCGCATGCTCCCGGCCGCCATGGCGGCCGCGGGAATTCGATTGGCGAAGAAACTCTACCAATAGCCACATAAATACATTAAGA<br>CGAGGATAAATAATCAGTGATACAAAAATGTCTCGCTTTCAAATAATAAATAGGTATCGTTTAGTCCGATGCGATCCTTACGTTACGGAGGAATTCATAAATTAACTCGTCGGAAATAAG<br>CTGGGCGCTCACAGCTGCTCTAACACACCTCAGCGCGACGCGCTGCTACCTCTACGTGATCCCTACGACTTACCTCGGGCACACCGCTACCAGCTACCAGTCCCCGGGTCGCGGCGTATT<br>GCACTGCCGTGCTCCGAGCAGCTCGGAGCAGCAAAAGGGGCGGCCCCGGGGGGCACGCACCCGCCCCGTCGGCCCCGTCCCGTCGGAGGAGTGAGGTAATAGAGGCGAGCCCGCTCCG<br>CTCGCCGGCGCCGGCCCGGCCGCTACAGGTGCGTGAGCTTGGGGGCCTCCTGCAGCCGTCCCTGAGACGCGTGGTGCGAGGAGAGGTCCGTGTAGGTGGGGTGGGGGTCGCAAGGTCCG<br>TGGTGGTGGCCGCCGGGGATCTGCGCGGCGCAGCTGTAGTCCACGCTGGCGGAGGAGGGGTGCTGCAGGTGGCCCAGGTTGAAGACGGGCCCCGAGGCCGGCATCGAGTCCACGAAGT<br>TGCAATCACTAGTGAATTCGCGGCCGCCTGCAGGTCGAC
HOXD4	Lab (AH)	NcoI / Sp6	ATTTAGGTGACACTATAGAATACTCAAGCTATGCATCCAACGCGTTGGGAGCTCTCCCATATGGTCGACCTGCAGGCGGCCGCGAATTCACTAGTGATTCCAAAAGCATCACACTCGCTG<br>TGCGGGATAAAAAGACAATTAAAAGCAAAATAATAATAATAAAATCCCAGAATAAAGCATCAAACCTTCATTTTCAAATCCCAAACTGAAACACAGGACAGTTTCATTTTCAATGATTT<br>ATTACTTTTTATGGACAGAAGGTCTCGTTTAAGACTTTCCCAATTCCTACTGCCTGTATGATGCTTTTTTTTCCCCCCTTTCTTTCTGTGCTAGCGAGAGGCAAATAAAATAAAGTCCGGAG<br>TAGCGTCATTCAGTCTCCTTTGTACCACAGTACCTTACGGAGAGATGGCAGTTTCTCGAGTCCTTGGCTGCTACACGCAGGTTCTGCTTGGATAGTGTCACTTATGTACAGTACTGGTCAG<br>AATCGAAGGATGAAATGGAAAATTTCACCTCTTCTATAAAGTTGTCAAGTCAGTCTGATGGTCCTTGGGAACAGTCTGTAAGTGGGGGTTAGAAGCGGAGGAGGAAGACCTGCCCTTAG<br>TGTTGGGCAGTTTGTGGTCTTTTTTCCACTTCATTCTTCTGTTCTGAAACCAGATCTTGATCTGGCGCTCAGAGAGACACAAAGTGTGCGCTATCTCGATACGGCGGCGCCGGGTCAGGTA<br>CCTGTTAAAATGAAATTCTTTTTCCAGTTCTAGGACTTGCTGCCTAATCGAATTCCCGCGGCCGCCATGG
HOXD8	Lab (NY)	NotI / T3	ATTAACCCTCACTAAAGGGACTAGTCCTGCAGGTTTAAACGAATTCGCCCTTTGTCGTCTTCCAGATCATGTGTTTCCTTTTTGGCCTCCCCTTCCTTTCCCTCCTGCTTGGAAGCGGGGAA<br>TTTGTTCTTGTTGTTCTCCTTTTTCCACTTCATCCTCCTGTTCTGAAACCAGATCTTTACCTGCCGCTCGGTGAGTCCTAGGGCGTGGGACACCTCGATTCGTCTCTTCCTGTCAGATAGGGG<br>TTAAAGAGAAACTCCTTCTCCAGCTTCAGCGTCTGGAAGCGGCTGTACGTCTGCCGCCCTCTCCTCCTCCCGGGCGCTGCTTGCGGTCTCATCCAGGGAAACATTTGAGCGGGAGACGAG<br>CTCTGATTTAAGTGGTCTGGTTCCTCGCCAATATTAGCACTGGACGATTTACAGTCAGGATATTGTACCAGCTCGGCCTCTTGCTGTGTCGTAAAAATCTGCTGTCTCTCGGGCGCGAAGT<br>GACAGTCGTAGTACGGGGCGGGCAGAGGCTCCCCCGCCGCCACCGCCGCCGCAGCCGCAGCCGCCGCCTTGTACTTGGAGTAGAGCGGGTTGACAAAGTAAGGGC
HOXD9	(Burke et al., 1995)	ClaI / T3	ATTAACCCTCACTAAAGGGAACAAAAGCTGGAGCTCCACCGCGGTGGCGGCCGCTCTAGAACTAGTGGATCCCCCGGGCTGCAGGAATTCGATGGGAAGGGCTCGATCCAGGAGCGCAC<br>GTAGCGGCCGGCGTCGGCTGCCCCTAAGTGGGGCTGGTGCATGTAGGGGTGGTAGATCCCGGTCATGGCCGCGGGCGGCTGCGGGTGAACCGTGGACCAGGAGGTGGAGAACACGGTG<br>GATTTTGGTGCAAAGCTACAGGAGGAAAAATCGGAACTCTCTGCCACACCTGATGGCCTGGAGGTCGCACTGTGACCCCCCTGGACGAATCTACTCCCGTAAACTTCTTCGCTTTCATGG<br>CCTATGAGAGAATCGACATAATAGTTACTTATGGTGCCACTAGACGACATTGCGGCCGCGAATTCGATATCAAGCTTATCGAT
HOXD11	(Burke et al., 1995)	EcoRI / Sp6	GATTTTTTTTTTTACATTTTTTCCTTTCTTTTTTTTTTCCTTTCTTTTTTTTTTTTTCTTTCTTTTTTTCCTCCTCAAAACAAGGGATTTCCAGTGAAGTATTGGAGTCGATCTCTGTTCAGTTTC<br>TTTTCTTTCATCCTTCGATTCTGGAACCAGATTTTAACTTGCCGGTCAGTGAGGTTGAGCATTCTGGACAACTGCAGTCTTTTCTCTTTGTTTATGTACACGTTGAAGAAAAACTCACGTTC<br>CAGTTCTCTTATCTGATATTTGGTATAGGGACACCTCTTTTTCCTCGATCGCTGAGGAACTGCTGAACTGTTGCTCTTCTCTGCAACAGCCTCACCGGGAGGGGAAGCGGCGCC
SHH	Lab (AH)	NcoI / Sp6	TGGACGAAATGCTGCTGTTGACAAGAATTCTCTTGGTGGGCTTCATCTGCGCTCTTTTAGTCTCCTCTGGGCTGACTTGTGGACCAGGCAGGGGCATTGGAAAAAGGAGGCACCCCAAAA<br>AGCTGACCCCGTTAGCCTATAAGCAGTTTATTCCCAATGTGGCAGAGAAGACCCTAGGGGCCAGTGGAAGATATGAAGGGAAGATCACAAGAAACTCCGAGAGATTTAAAGAACTAAC<br>CCCAAATTACAACCCTGACATTATTTTTAAGGATGAAGAGAACACGGGAGCTGACAGACTGATGACTCAGCGCTGCAAGGACAAGCTGAAcGCCCTGGCGATCTCGGTGATGAACCAGT<br>GGCCCGGGGTGAAGCTGCGGGTGACCGAGGGCTGGGACGAGGATGGCCATCACTCCGAGGAATCGCTGCACTACGAGGGTCGCGCCGTGGACATCACCACGTCGGATCGGGACCGCAG<br>CAAGTACGGAATGCTGGCCCGCCTCGCCGTCGAGGCCGGCTTCGACTGGGTCTACTACGAGTCCAAGGCGCACATCCACTGCTCCGTCAAAGCAGAAAACTCAGTGGCAGCGAAATCAG<br>GAGGCTGCTTCCCTGGCTCAGCCACAGTGCACCTGGAGCATGGAGGCACCAAGCTGGTGAAGGACCTGAGCCCTGGGGACCGCGTGCTGGCTGCTGACGCGGATGGCCGGCTGCTCTAC<br>AGCGACTTCCTCAC

AH: Aurélie Hintermann ; JZ: Jozsef Zakany (University of Geneva, Switzerland); NY: Nayuta Yakushiji-Kaminatsui (Tokyo University of Science, Japan)

Development • Supplementary information

Table S4. List of ChIP-seq experiment batches

Sample	Machine	Run	Experimenter	Study
ChIP_H3K27ac_HH18_PT_rep1	K00341	187	AH	This study
ChIP_H3K27ac_HH18_PT_rep2	K00341	187	AH	This study
ChIP_H3K27ac_HH35_DS_rep1	K00341	187	AH	This study
ChIP_H3K27ac_HH35_DS_rep2	K00341	187	AH	This study
ChIP_H3K27ac_HH35_FB_rep1	K00341	187	AH	This study
ChIP_H3K27ac_E125_DFL_rep1	HWI-ST865	262	LB	GSM2713703
ChIP_H3K27ac_E125_FB_rep1	HWI-ST865	262	LB	This study
ChIP_H3K27ac_E125_PFL_rep1	HWI-ST865	262	LB	GSM2713704
ChIP_H3K27ac_E85_PT_rep1	HWI-ST865	366	LB	This study
ChIP_H3K27ac_E85_PT_rep2	HWI-ST865	402	LB	This study
ChIP_H3K27ac_E125_WP_rep1	HWI-ST865	478	AH	This study
ChIP_H3K27ac_HH28_DFL_rep1	HWI-ST865	487	NY	GSM3182462
ChIP_H3K27ac_HH28_PFL_rep1	HWI-ST865	487	NY	GSM3182459

AH: Aurélie Hintermann
LB: Leonardo Beccari
NY: Nayuta Yakushiji-Kaminatsui

References

Allais-Bonnet, A., Hintermann A., Deloche, M.-C., Cornette,R., Bardou, P., Naval-Sanchez, M., Pinton, A., Haruda, A., Grohs, C. and Zakany, J. et al. (2021).Analysis of polycerate mutants reveals the evolutionary co-option of HOXD1 for horn patterning in Bovidae. *Mol. Biol. Evol.* **38**, 2260-2272. doi:10.1093/molbev/msab021

Burke, A. C., Nelson, C. E., Morgan, B. A. and Tabin, C. (1995). Hox genes and the evolution of vertebrate axial morphology. *Development* **121**, 333-346. doi:10.1242/dev.121.2.333

Condie B. G. and Capecchi, M. R. (1993). Mice homozygous for a targeted disruption of Hoxd-3 (Hox-4.1) exhibit anterior transformations of the first and second cervical vertebrae, the atlas and the axis. *Development* **119**, 579-595. doi:10.1242/dev.119.3.579

Dollé, P., Izpisúa-Belmonte, J. C., Falkenstein, H., Renucci, A. and Duboule D. (1989). Coordinate expression of the murine Hox-5 complex homoeobox-containing genes during limb pattern formation. *Nature* **342**, 767-772. doi:10.1038/342767a0

Echelard, Y., Epstein, D. J., St-Jacques, B., Shen L., Mohler, J., McMahon, J. A. and McMahon, A. P. (1993). Sonic hedgehog, a member of a family of putative signaling molecules, is implicated in the regulation of CNS polarity. *Cell* **75**, 1417-1430. doi:10.1016/0092-8674(93)90627-3

Featherstone, M. S., Baron, A., Gaunt, S. J., Mattei, M. G. and Duboule, D. (1988). Hox-5.1 defines a homeobox-containing gene locus on mouse chromosome 2. *Proc. Natl. Acad. Sci. USA* **85**, 4760-4764. doi:10.1073/pnas.85.13.4760
