# Supplementary material for: Developmental and evolutionary comparative analysis of a regulatory landscape in mouse and chicken
Source: Development. 2022 Jun 30;149(12):dev200594. doi: 10.1242/dev.200594 (PMC9307994; doi:10.1242/dev.200594)
Supplement: Reviewer comments [file develop-149-200594_review_history.pdf]

Developmental and evolutionary comparative analysis of a regulatory landscape in mouse and chicken

Aurélie Hintermann, Isabel Guerreiro, Lucille Lopez-Delisle, Christopher Chase Bolt, Sandra Gitto, Denis Duboule and Leonardo Beccari
DOI: 10.1242/dev.200594

Editor: Benoit Bruneau

Review timeline

Original submission:	29 January 2022
Editorial decision:	28 February 2022
First revision received:	22 May 2022
Accepted:	23 May 2022

Original submission

First decision letter

MS ID#: DEVELOP/2022/200594

MS TITLE: DEVELOPMENTAL AND EVOLUTIONARY COMPARATIVE ANALYSIS OF A REGULATORY LANDSCAPE IN MAMMALS AND BIRDS

AUTHORS: Aurelie Hintermann, Isabel Guerreiro, Christopher Chase Bolt, Lucille Lopez-Delisle, Sandra Gitto, Denis Duboule, and Leonardo Beccari

I have now received all the referees' reports on the above manuscript, and have reached a decision. The referees' comments are appended below, or you can access them online: please go to BenchPress and click on the 'Manuscripts with Decisions' queue in the Author Area.

As you will see, the referees express considerable interest in your work, but have some significant criticisms and recommend a substantial revision of your manuscript before we can consider publication. If you are able to revise the manuscript along the lines suggested, which may involve further experiments, I will be happy receive a revised version of the manuscript. The main requested experiment is cross-species enhancer examination in mouse. Otherwise it appears text revisions would be sufficient. Your revised paper will be re-reviewed by one or more of the original referees, and acceptance of your manuscript will depend on your addressing satisfactorily the reviewers' major concerns. Please also note that Development will normally permit only one round of major revision.

We are aware that you may be experiencing disruption to the normal running of your lab that make experimental revisions challenging. If it would be helpful, we encourage you to contact us to discuss your revision in greater detail. Please send us a point-by-point response indicating where you are able to address concerns raised (either experimentally or by changes to the text) and where you will not be able to do so within the normal timeframe of a revision. We will then provide further guidance. Please also note that we are happy to extend revision timeframes as necessary.

Please attend to all of the reviewers' comments and ensure that you clearly highlight all changes made in the revised manuscript. Please avoid using 'Tracked changes' in Word files as these are lost in PDF conversion. I should be grateful if you would also provide a point-by-point response detailing

© 2022. Published by The Company of Biologists under the terms of the Creative Commons Attribution License (https://creativecommons.org/licenses/by/4.0/).

how you have dealt with the points raised by the reviewers in the 'Response to Reviewers' box. If you do not agree with any of their criticisms or suggestions please explain clearly why this is so.

Reviewer 1

Advance summary and potential significance to field

Summary:
In this study, the authors focus on the cis-regulatory elements (CREs) and related modalities responsible for species-specific divergence of HoxD expression in skin appendage development (mouse VPs and chicken FPs) and posterior trunk elongation, the latter a process highly conserved across amniotes. After demonstrating the relevant HoxD expression patterns in mouse and chicken skin primordia, the authors define the species-specific chromatin interaction profiles across the HoxD locus and neighboring TADs.
Interestingly, Capture-HiC and 4C-seq from posterior trunk cells revealed a largely conserved regulatory topology at mouse and chicken HoxD loci, and indicated preferential interactions of HoxD1, 4 and 9 genes specifically with segments of the telomeric regulatory domain (D1, D4, D9 intervals). Assuming conserved regulatory topology across tissues, the authors used H3K27ac profiling in mouse VPs, chicken FPs and embryonic trunks to predict species-specific enhancer modules underlying HoxD expression in these structures, and find highest densities of putative enhancer elements in domains hallmarked by increased tissue-specific contact frequencies, as indicated by 4C-seq. The authors then characterize individual HoxD1 enhancers using a functional approach relying on (BAC) LacZ reporter and large deletion analysis (using previously established BACs/deletion alleles) in mouse embryos and identify several regulatory modules likely responsible for HoxD1 regulation in VPs and somites. Subsequently, the authors include additional H3K27ac datasets (from other studies) to perform an integrative analysis to delineate genome-wide divergent and conserved tissue-specific activities of putative enhancer modules underlying mouse and chicken development. Interestingly, they find that individual CREs most commonly harbor species-specific activity signatures and that increased sequence conservation in candidate enhancers may reflect higher evolutionary pressure due to pleiotropic activities.

Novelty and Impact:
This study provides important novel insight into the regulatory dynamics and level of genomic conservation underlying evolutionary diversification of specific embryonic structures/compartments. By focusing on the HoxD locus the authors comprehensively assess the complexity and functional segmentation of extensive regulatory domains linked to a multi-gene cluster with critical functions in a diverse range of tissues. Making use of this framework and an extensive available genetic toolbox (for critical in vivo validation), the authors are able to provide an estimate of the degree of evolutionary regulatory novelty underlying phenotypic diversification at the HoxD locus and genome-wide. While VPs and FPs have evolved into divergent structures in mice and chicken, they still rely on conserved gene networks. This study suggests that despite the similarities of gene expression patterns orchestrating the development of related structures in distinct classes of vertebrates, the underlying regulatory activities might be more divergent as commonly assumed. In fact, such a regulatory divergence in species-specific (mouse-chicken) enhancer activities has also been recently observed in comparative analyses focusing on the developing limbs (Jhanwar et al., 2021: PMID:
34584102).

Quality:
The authors make use of a comparative approach involving spatial gene expression analysis, chromatin conformation capture and H3K27ac profiling in specific tissues of mouse and chicken embryos to comprehensively delineate the chromatin and cis-regulatory architectures underlying control of the HoxD cluster during development and evolution. Overall, this study provides a significant advance in our understanding of the gene regulatory mechanisms underlying evolutionary diversification of highly specified structures/tissues. The manuscript is well written, and the figures and illustrations are of good quality. I consider the techniques applied, analyses performed, and results obtained technically and biologically robust. While the genome-wide conclusions are mainly relying on the use of H3K27ac as a proxy for in vivo enhancer activities, the authors provide an example of the importance of in vivo enhancer characterization by using

© 2022. Published by The Company of Biologists under the terms of the Creative Commons Attribution License (https://creativecommons.org/licenses/by/4.0/).

reporter transgenics and related deletion analysis, which leads to the identification of a novel mouse VP and somite enhancer, respectively.

Comments for the author

The following points should be addressed before publication:

Major comments:
- Given the significance of the D1, D4 and D9 intervals in this study as “preferentially interacting regions”
for the respective HoxD genes, it remains unclear how exactly the extensions of these regions were determined (as based on manual annotations). A more precise rational based on (quantitative) interaction profiles, CTCF enrichment and/or chromatin marks would be preferable to infer such domains.
- Use of MARs as genomic regions enriched for highest H3K27ac enrichment (after subtraction of a control signal of an un-related tissue) appears as a new feature that seems useful for ranking tissue- or species-specific enhancer elements. Did the authors exclude promoter signals for this approach? Also, the rational for using sliding windows of 10kb every 2kb should be described in more detail.
- The overlap of accessible chromatin with generally more broadly distributed H3K27ac has now emerged as standard signature of transcriptional enhancers. To verify the technical specificity of the H3K27ac-focused approach chosen, the authors should demonstrate that their H3K27ac ChIP-seq signals from chicken and mouse samples are reproducibly overlapping open chromatin regions.
- Related to line 418: To support the claim that a majority of evolutionary conserved CREs may show divergent activity patterns in the respective species (as indicated in Fig. 5B, C; and also suggested by other studies, e.g. Villar 2015), this present study would gain from comparative transgenic reporter analyses (in mouse embryos) assessing mouse/chicken versions of conserved elements with predicted divergent enhancer potential.

Minor comments:
- While the title of this study points to the findings presented, it is too generalized in my opinion. A focus on the HoxD regulatory landscape and/or the tissues characterized would be preferable.
- Figure S1A: the authors should explain the meaning of 1WGD and 2WGD in the legend
- Figures 3/S4: “H3K27ac” should be added to the respective tracks as a label
- Figure S4, panel E, chicken: the “embryo schematic” is missing.

<u>Reviewer 2</u>

Advance summary and potential significance to field

Changes in gene regulatory networks greatly contribute to evolution, so it is important to understand variations in regulatory landscapes that contribute to morphological differences between different species. In this study, Hintermann and colleagues compared the regulatory landscape of the HoxD gene cluster between chicken and mice in two distinct developmental programs, the elongation of the posterior trunk, which is highly conserved, and skin appendages which are thought to evolve independently in chicken and mice. The authors first show that there are expression differences between different HoxD genes in both murine vibrissae (VP) and chicken feather primordia (FP), suggesting that these independently evolved skin appendages might be controlled by different subsets of HoxD genes and CREs.
In contrast, the expression of HoxD genes in the developing posterior trunk was largely conserved between different species. The authors then performed epigenomic profiling of these two pairs of tissues in chicken and mice using Capture-HiC (only for trunks) and H3K27ac ChIP-seq. They also complemented their study with analysis of a series of deletions and BAC transgenes spanning the HoxD locus to determine requirements of different HoxD parts for HoxD genes expression in mouse skin and trunk.

© 2022. Published by The Company of Biologists under the terms of the Creative Commons Attribution License (https://creativecommons.org/licenses/by/4.0/).

Comments for the author

The quality of data is perhaps the strongest part of the paper. In situ hybridizations to analyze gene expression between two species, trunk Hi-C data and comparative H3K27ac data are of very good quality. This data will benefit developmental biologists studying these tissues/processes, although experiments to assess skin appendages were performed on bulk skin tissues, which will significantly dilute the signal from skin appendage precursors. The study is very descriptive in nature with little functional validation. While the study contains some genome-wide datasets, the authors focused only on the HoxD locus. The rationale behind choosing the HoxD locus is unclear. Most previous studies showed that HoxC genes are the critical regulators for hair development (including vibrissae and feather primordia; e.g., PMIDs: 33199643, 9420327, 9389453).

Comments to the authors:
1. Fig 3A: H3K27ac ChIP-seq experiments to assess VP and FP regulatory landscapes were performed from bulk tissue, which may contribute to the weaker VPs and FP-specific signals on both HoxD genes and their regulatory elements in Fig3A.
2. How accurate are comparisons between equivalent developmental stages between chicken and mouse embryos?
3. Fig. 3A: H3K27ac is only predictive of divergent enhancer activity. Is there a way to functionally validate divergent activity? For example, can the chicken D1 region drive the expression in VP?
4. Fig. S3. Proper statistical analysis is lacking and needs to be performed to assess the difference in interactions frequencies between 4C datasets. The differences among interaction frequencies of D1, D4, D9 are subtle, and the difference between HoxD1 and HoxD4 may be mainly due to D1 being close to the bait region for HoxD1.
5. Fig5B: The authors claim that “the enhancer profiles of CNEs were more similar between different tissues of the same species than between the same tissues in mouse and chicken”, Could that be due to the experimental batch effects? It’s a well-known caveat of bulk analysis between different tissues/species (see PMID: 26236466).
6. Fig. 2: Change the color scale to make the TAD plot interactions more visible.
7. The signal on MARs in the lower panel of Fig3A appears weak -> try to rescale the y-axis?
8. Line 311, there should be two p-values (mouse and chicken).

First revision

Author response to reviewers' comments

Reviewer 1

Comments for the Author:
The following points should be addressed before publication:

Major comments:

R1C1
-Given the significance of the D1, D4 and D9 intervals in this study as “preferentially interacting regions” for the respective HoxD genes, it remains unclear how exactly the extensions of these regions were determined (as based on manual annotations). A more precise rational based on (quantitative) interaction profiles, CTCF enrichment and/or chromatin marks would be preferable to infer such domains.

The definition of the interacting regions was based on 4C-seq interaction profiles in mouse and sequence conservation. The regions are delimited by CNEs to be able to transpose them to the chicken genome. CNE331 was chosen for the 5’ border of region D1 as it is the only CNE located in the region that spans from the 3’ of the *HoxD* cluster to the promoter of *Mtx2*. At the other extremity, the 3’ border of D9 region was set to CNE382, where the *Hoxd9* signal goes back to the same level as *Hoxd4* (Figure S3A). The limits between two regions (“D1/D4” and “D4/D9”) were

© 2022. Published by The Company of Biologists under the terms of the Creative Commons Attribution License (https://creativecommons.org/licenses/by/4.0/).

established based on 4C scores normalized by the number of mapped reads. To obtain the relative distribution of contacts across our region of interest (i.e., from CNE331 to CNE382), we first normalized 4C scores by the total score of the whole region. We then computed the cumulative sum of the difference between the normalized scores of two viewpoints (Figure S3C). Accordingly, the delimitation between region D1 and region D4 was set to CNE346, which is the closest conserved element to the minimum value of the cumulative sum using the score difference *Hoxd4-Hoxd1* and thus corresponds to the end of the genomic segment where *Hoxd1* values are higher than those of *Hoxd4*. Similarly, the D4/D9 border was set to CNE364, which corresponds to the genomic location for which the cumulative sum of the score difference *Hoxd9-Hoxd4* is minimum and where *Hoxd9* values become higher than those of *Hoxd4*. The limits of the D-regions were then transposed to the chicken genome using corresponding CNEs. Finally, the normalized 4C scores for each viewpoint were quantified in each region and show that most viewpoint's scores are higher in the region annotated as their preferentially interacting region (i.e., *Hoxd1* / *HOXD1* scores are higher in mouse and chicken D1-regions, respectively)."

We have now added this paragraph to the material and methods section under: "Annotation of the D-regions". Accordingly, we have added a panel **C** to Figure S3, where the cumulative sums of the normalized score differences between two adjacent viewpoints are plotted, showing the genomic location where the shift in preferential interaction frequencies occurs. We have also added a panel **D** to Figure S3, showing the relative distribution of normalized scores for each viewpoint within each region.

R1C2

-Use of MARs as genomic regions enriched for highest H3K27ac enrichment (after subtraction of a control signal of an un-related tissue) appears as a new feature that seems useful for ranking tissue- or species-specific enhancer elements. Did the authors exclude promoter signals for this approach? Also, the rational for using sliding windows of 10kb every 2kb should be described in more detail.

Yes, this is an important question. We could have adapted the algorithm to remove promoter signals from the ChIP-seq data, but we preferred not to. We reasoned that promoter activities belong to regulatory activities, and that they are biologically relevant as the signal is tissue specific (i.e., we cannot exclude that the activity of a promoter may enhance the activity of another promoter nearby, a phenomenon that has been documented in the literature). We thus choose to consider the regulatory region as a whole region, including its tissue-specific promoter activities.

The parameters of the size of the sliding windows impact mostly the speed of computation. The region detection is robust to different parameters (see Figure R1C2 at the end of this document). We set the window size to 10kb for simplification: it is safe to assume that the threshold score would not be reached within 10 kb (10kb is small enough), as the minimum proportion is 30% of the region, and the smallest MAR is 400 kb. On the other hand, the computation is performed in reasonable time (10kb is big enough).

We choose 2kb for the size of the step, which defines the "resolution" of the MAR, as it is in the range of the sizes we could expect for regulatory elements. We tried with 1kb and with 5kb, which did not change the biological conclusion (same region, small shift in both the start and the end of coordinates; see Figure R1C2).

R1C3

-The overlap of accessible chromatin with generally more broadly distributed H3K27ac has now emerged as standard signature of transcriptional enhancers. To verify the technical specificity of the H3K27ac-focused approach chosen, the authors should demonstrate that their H3K27ac ChIP-seq signals from chicken and mouse samples are reproducibly overlapping open chromatin regions.

We agreed with this comment and thus generated an ATAC-seq dataset for mouse and chicken forebrain (FB), posterior trunk (PT) and skin (VP in mouse and FP in chicken) tissues, in two replicates.

© 2022. Published by The Company of Biologists under the terms of the Creative Commons Attribution License (https://creativecommons.org/licenses/by/4.0/).

We have now complemented Figure 4F with ATAC-seq coverage tracks to refine our enhancer candidate (EC) selection. EC1 and EC3 are ATAC-seq positive in VPs. In PT, EC2 is H3K27ac-positive and the ATAC-seq data shows that it is also accessible. This nicely correlates with our transgenic results where EC1 is positive in E12.5 VPs and EC2 in E9.5 PTs. To make it reader-friendly, we added stars on the top of the arrowheads that are showing H3K27ac-positive (black) or negative (white) ECs, in order to visualize which ones are also open.

ATAC-seq coverage tracks were added to Figure S4 D and E as an indication for the levels of chromatin accessibility of those H3K27ac peaks considered. We adapted the peak tracks in order to keep only H3K27ac peaks that were overlapping ATAC-seq peaks. The correspondence between ATAC and H3K27ac enrichment was generally good, with some exceptions which could be due for example to elements that are not yet or no longer active, or that may function as repressors. It could also simply be due to technical specificities of the ChIP versus ATAC-seq protocols, as they are based on different principles and experimental procedures (i.e., detection of histone modifications requires having nucleosomes while chromatin accessibility is detectable also in nucleosome free-regions).

Finally, to demonstrate that the H3K27ac ChIP-seq peaks quantified in Figure 5 reproducibly overlap with open chromatin regions, we produced a new supplementary figure (Fig. S5). In panel A, we quantified the proportion of H3K27ac ChIP-seq peaks that overlap ATAC-seq peaks in matching tissues. A large majority of ChIP-seq peaks overlap with an ATAC-seq peak present in either one or both replicates, which we call “open H3K27ac peaks’ for the sake of simplicity. Panels B and C were produced using the same approaches as for panels B and C of Figure 5, yet this time we kept enhancer-CNEs that overlapped ATAC-seq peaks (referred to as “open enhancer-CNEs”). The clustering of open enhancer-CNEs on the heatmap, or their distribution in the different sets of Euler diagrams, are perfectly comparable to the case when we use all ChIP-seq peaks (overlapping or not with ATAC-seq peaks).

R1C4
-Related to line 418: To support the claim that a majority of evolutionary conserved CREs may show divergent activity patterns in the respective species (as indicated in Fig. 5B, C; and also suggested by other studies, e.g. Villar 2015), this present study would gain from comparative transgenic reporter analyses (in mouse embryos) assessing mouse/chicken versions of conserved elements with predicted divergent enhancer potential.

We agree that the work ‘would gain’ from adding some transgene reporter assays (which my laboratory has carried out heavily and for many years). We nevertheless decided not to invest into this approach, for various reasons. Transgenesis assays are known to produce a number of caveats for the interpretation of enhancer activity besides being highly demanding in terms of workload and in the number of animals, particularly if targeted (single-copy) transgenesis approaches are used which is the gold standard these days. Besides the fact that we do not have the possibility to perform transgene reporter essays in chicken (we do not trust the currently used methodologies used in chicks) and thus the impossibility to analyze whether a chick sequence is able to drive reporter gene expression in chick -which in any case would not guarantee that its activity would be the same in the mouse (and vice-versa)- the current Swiss law on laboratory animals protection would require to file a specific request for these experiments and an explanation as to how absolutely necessary they would be in the context of the 3R principles, an explanation I do not feel ready to give. Such an authorization would take another 6 to 8 months.

R1C5s
Minor comments:

-While the title of this study points to the findings presented, it is too generalized in my opinion. A focus on the HoxD regulatory landscape and/or the tissues characterized would be preferable.

We understand this comment yet we find it difficult to reduce it to the tissues analyzed. Regarding the *HoxD* cluster, it is flanked by essentially three regulatory landscapes (C-DOM, T-DOM subTAD-1 and T-DOM subTAD-2). A complete title would be:

© 2022. Published by The Company of Biologists under the terms of the Creative Commons Attribution License (https://creativecommons.org/licenses/by/4.0/).

DEVELOPMENTAL AND EVOLUTIONARY COMPARATIVE ANALYSIS OF ENHANCERS SPECIFIC FOR VIBRISSAE, SOMITES AND FEATHER BUDS FOUND IN ONE OF THE THREE HOXD REGULATORY LANDSCAPES IN MAMMALS AND BIRDS.

We prefer to keep our title which exactly reflects what is in the paper. The higher precision asked by this expert is found in the abstract. Also, a good part of the paper goes genome-wide at the end.

-Figure S1A: the authors should explain the meaning of 1WGD and 2WGD in the legend.
-Figures 3/S4: "H3K27ac" should be added to the respective tracks as a label
-Figure S4, panel E, chicken: the "embryo schematic" is missing.

Yes, this is well taken and we modified the figures accordingly.

Reviewer 2

Comments for the Author:

The quality of data is perhaps the strongest part of the paper. In situ hybridizations to analyze gene expression between two species, trunk Hi-C data, and comparative H3K27ac data are of very good quality. This data will benefit developmental biologists studying these tissues/processes, although experiments to assess skin appendages were performed on bulk skin tissues, which will significantly dilute the signal from skin appendage precursors. The study is very descriptive in nature with little functional validation. While the study contains some genome-wide datasets, the authors focused only on the HoxD locus. The rationale behind choosing the HoxD locus is unclear. Most previous studies showed that HoxC genes are the critical regulators for hair development (including vibrissae and feather primordia; e.g., PMIDs: 33199643, 9420327, 9389453).

We are sorry that the justification of this work was not exposed clearly enough. We are of course aware of these studies (one of us is a corresponding author of the first paper mentioned). The idea was not to study the regulation of tegument enhancers but, more generally, how such enhancers relate to one another between species. To carry out such a work, one needs to start with 'regulatory landscapes' that are well characterized in two different species and nothing like this exists with the *HoxC* cluster, while *HoxD* is certainly amongst the best characterized locus in terms of multiple and long-range regulations (along with globins). We tried to make this point clearer.

Comments to the authors:

R2C1
1. Fig3A: H3K27ac ChIP-seq experiments to assess VP and FP regulatory landscapes were performed from bulk tissue, which may contribute to the weaker VPs and FP-specific signals on both HoxD genes and their regulatory elements in Fig3A.

Yes. We show that *Hoxd* genes are expressed in primordia but not in the skin around, so while the signal is diluted, it remains specific. We tried to "punch" out the follicles from the skin, but it did not work for obvious reasons. Extracting individual hair or feather follicles from embryonic skin is hardly feasible.

R2C2
2. How accurate are comparisons between equivalent developmental stages between chicken and mouse embryos?

This is a valid question of course, and a difficult comparison to realize. We first approximated the correspondence of developmental stages between mouse and chicken by referring to Carnegie stage comparisons tables. We then refined those approximations using Hamburger Hamilton

© 2022. Published by The Company of Biologists under the terms of the Creative Commons Attribution License (https://creativecommons.org/licenses/by/4.0/).

descriptions and images, as well as by using our own morphological landmarks.

For example, for E9.5 posterior trunks, the forelimb bud (FLB) should be small but visible, the hindlimb bud should not be present or very small, the embryo is cut below FLB. Given the size of the embryonic trunk at E9.5, the number of animals necessary to do an H3K27ac ChIP-seq is high and we thus decided to use pre-existing datasets from the lab that were collected at E8.5, a stage when the expression of *Hoxd1* is similar to E9.5. In this case, embryos were cut at the level of the 2nd to 4th pair of somites.

For hair and feather follicle placodes, we dissected pieces of skin at a stage when the outgrowth/down growth of the VP/FP placode into a peg is not distinguishable. We assessed by ISH the expression of *Shh* (a marker of the early placode) and *Hoxd* genes at the selected stages (FigS1B, FigS2A).

Here we may add that the various parts of each embryo may not develop with the same ‘relative speeds’ and hence a proper adjustment may simply be theoretically impossible to realize, for example between a mouse and a human brain.

R2C3
3. Fig. 3A: H3K27ac is only predictive of divergent enhancer activity. Is there a way to functionally validate divergent activity? For example, can the chicken D1 region drive the expression in VP?

Yes, because H3K27ac is indeed predictive, we turned to genome wide analyses, to be able to claim that the functions of conserved sequences often diverge. The functional validation of enhancer candidates *via* transgenic assay without single-copy targeting (see above) provides only limited support and display high variability. Introducing series of candidates in a targeted locus is demanding in workload and in number of animals. Moreover, the robustness of the results is often even worst across species, for if we know how a chicken enhancer is ‘written’, we do not know if a chicken cell would read it the same way as a mouse cell. Our laboratory was the first to replace a mouse enhancer sequence by the fish counterpart by recombination (in the 1990’s) and we know too well how difficult it is to interpret these often-disappointing experiments, even when caried out at the same orthologous locus.

R2C4
4. Fig. S3. Proper statistical analysis is lacking and needs to be performed to assess the difference in interactions frequencies between 4C datasets. The differences among interaction frequencies of D1, D4, D9 are subtle, and the difference between HoxD1 and HoxD4 may be mainly due to D1 being close to the bait region for HoxD1.

The proximity effect is generally observed with high contacts around the bait, which exponentially decay in function of the distance to the viewpoint. Therefore, the only boundary that may be (and likely is) impacted by the proximity effect would be the 5’ border of the D1 region. We set it to CNE331 as it is the only conserved sequence in this location and we wanted to consider as much of the regulatory landscape as possible (CNE332 is located 64 kb more telomeric than CNE331). We could have set it to CNE332, which would better ensured very few proximity effects. However, we are not convinced that this is relevant, because we did not aim at defining highly precise limits of interactions. Instead, the main conclusion of this part of the work is the differential distribution of *Hoxd* promoter contacts within the T-DOM, which inversely correlates with their position in the cluster (3’ *Hoxd* genes contact preferentially more 5’ T-DOM regions), rather than the precise limits of each domain. Thus, variations in a D1 boundary region due to viewpoint distance correction would not change the conclusion (and is also affected by other parameters such as the sliding window size or the restriction enzyme used to produce the 4C library).

To clarify this point and our approach, we have added a paragraph in Material and Methods explaining in detail how each border was delimited using 4C-seq interaction profiles in mouse and sequence conservation. Then, we quantified the distribution of normalized 4C scores within each region, for each viewpoint and in both species (see our answer to R1C1 for further details).

© 2022. Published by The Company of Biologists under the terms of the Creative Commons Attribution License (https://creativecommons.org/licenses/by/4.0/).

R2C5

5. Fig5B: The authors claim that "the enhancer profiles of CNEs were more similar between different tissues of the same species than between the same tissues in mouse and chicken", Could that be due to the experimental batch effects? It's a well-known caveat of bulk analysis between different tissues/species (see PMID: 26236466).

To better evaluate the possibility that "the enhancer profiles of CNEs were more similar between different tissues of the same species than between the same tissues in mouse and chicken" could derive from experimental batch effects, we now provide the details of ChIP- seq experiment batches in a supplementary table S4. This table shows how samples from different batches cluster well together. For example, the mouse DFL sample is closer to the mouse PT sample than to the chicken DFL sample, despite having been processed by different members of the lab and at different times. However, we cannot demonstrate that samples of different species processed in the same batch would separate by species on the heatmap. Nevertheless, when we annotated the heatmap with both the different sequencing machines used, the various runs, the various experimenters as well as for which study each sample was processed, no clear association emerges between those variables, on the one hand, and the clustering obtained, on the other hand (see Figure R2C5). For this reason, we consider it as very likely that the major determinant of the clustering is the biological origin of the sample.

We can nevertheless not formally exclude that a batch effect related to the H3K27ac antibody efficiency in mouse versus chicken may exist, which could contribute to the sample clustering by species rather than by tissues. In any case, this would not impact the conclusion that evolutionarily conserved sequences display divergent regulatory activities in different species, since we find CNEs that are specifically active in mouse structures different than chicken structures or not active at all in the chicken tissues analyzed and vice-versa.

6. Fig. 2: Change the color scale to make the TAD plot interactions more visible.

This was done

7. The signal on MARs in the lower panel of Fig3A appears weak -> try to rescale the y-axis?

This was done

8. Line 311, there should be two p-values (mouse and chicken).

Sorry. This was done.

Annex:

Two figures for referees, described in the text above.

© 2022. Published by The Company of Biologists under the terms of the Creative Commons Attribution License (https://creativecommons.org/licenses/by/4.0/).

Hintermann et al.
R1C2_MARS_different_param

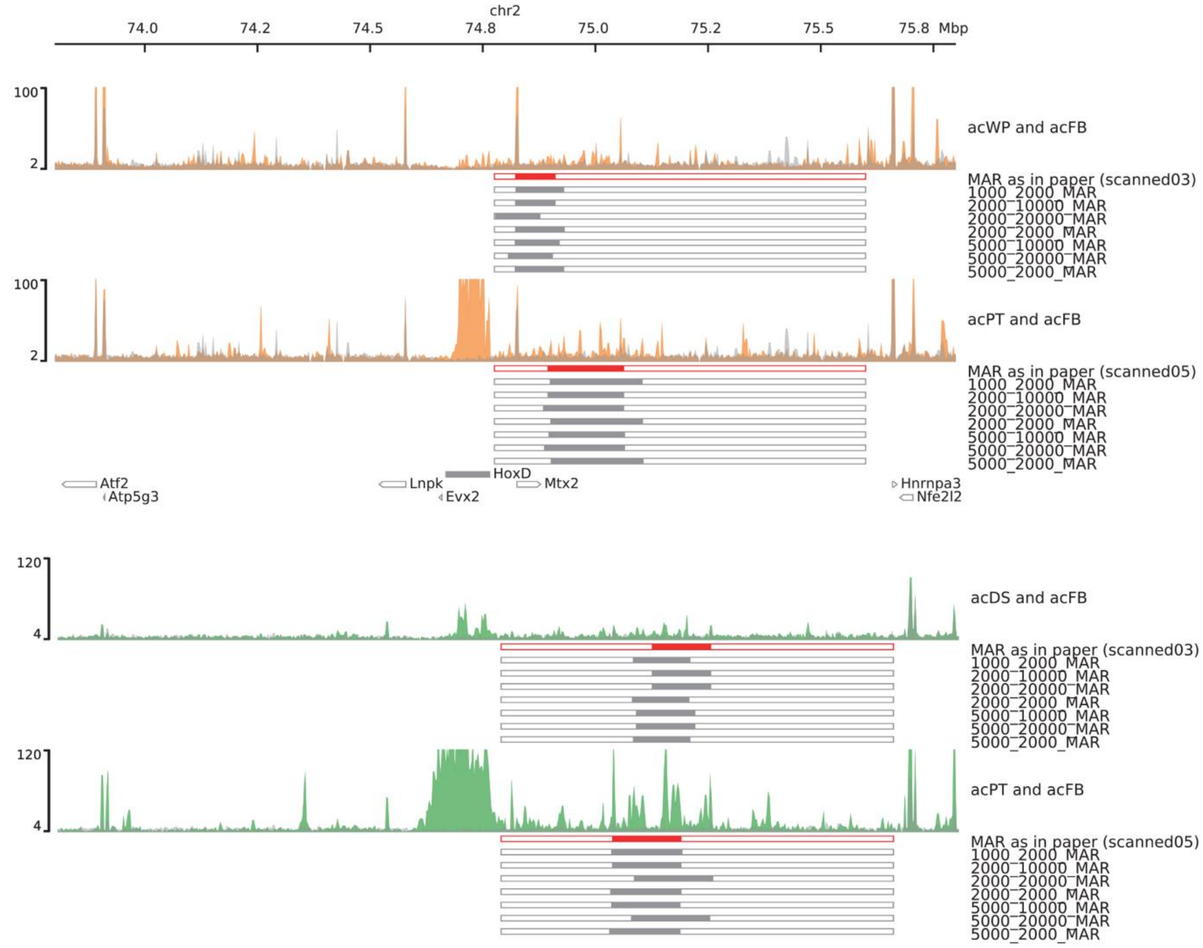

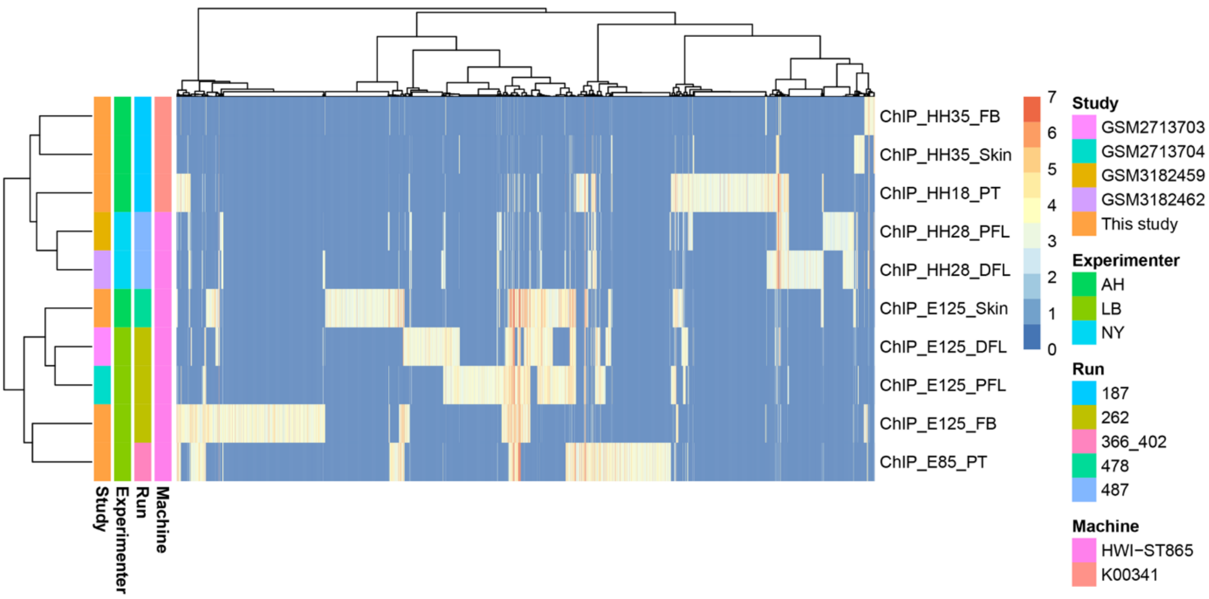

© 2022. Published by The Company of Biologists under the terms of the Creative Commons Attribution License (https://creativecommons.org/licenses/by/4.0/).

Second decision letter

MS ID#: DEVELOP/2022/200594

MS TITLE: DEVELOPMENTAL AND EVOLUTIONARY COMPARATIVE ANALYSIS OF A REGULATORY LANDSCAPE IN MAMMALS AND BIRDS

AUTHORS: Aurelie Hintermann, Isabel Guerreiro, Lucille Lopez-Delisle, Christopher Chase Bolt, Sandra Gitto, Denis Duboule, and Leonardo Beccari
ARTICLE TYPE: Research Article

I am happy to tell you that your manuscript has been accepted for publication in Development, pending our standard ethics checks.

© 2022. Published by The Company of Biologists under the terms of the Creative Commons Attribution License (https://creativecommons.org/licenses/by/4.0/).
